# Independent interferometric observable confirms black hole shadow in M87*

**DOI:** 10.1038/s41586-026-11241-9

**Published:** 2026-11-11

**Authors:** Samuel Lai, Nithyanandan Thyagarajan, O. Ivy Wong, Foivos Diakogiannis

**Affiliations:** 1 Space and Astronomy, Commonwealth Scientific and Industrial Research Organisation (CSIRO), Bentley, Western Australia Australia; 2 International Centre for Radio Astronomy Research (ICRAR)-M468, The University of Western Australia, Crawley, Western Australia Australia; 3 Technology, Commonwealth Scientific and Industrial Research Organisation (CSIRO), Kensington, Western Australia Australia

**Keywords:** General relativity and gravity, Compact astrophysical objects

## Abstract

A new epoch for direct visualization of accretion dynamics near the event horizon of a black hole began when the Event Horizon Telescope Collaboration synthesized an Earth-sized aperture with millimetre radio interferometry and published the first reconstructed image^1–4^ of a black hole shadow at the core of radio galaxy Messier 87 (M87). The image appeared as a ring-like structure, representing the gravitationally lensed and redshifted emission around the central supermassive black hole, M87*, located at the galactic nucleus. However, application of various data analysis and imaging heuristics resulted in divergent representations of M87* fine-scale features^3,5–7^, with some external analyses even challenging the fundamental ring-like morphology of the source^8,9^. Such difficulties arise as a consequence of the inherent sparsity of the very long baseline interferometry measurements, making interferometric image reconstruction an inadequately constrained problem. Moreover, the solution is sensitive to instrument calibration, post-processing algorithms and imaging hyperparameters. Here we apply an independent imaging algorithm that we have developed to the M87* dataset that is free from prescriptive morphological priors and founded on observables immune to instrument calibration errors. We reconstruct the black hole shadow image and unequivocally reproduce a ring-like morphology, with an angular diameter of 42.2 ± 1.5 μas corresponding to a black hole mass of 6.5 ± 0.5 billion solar masses assuming nominal scaling parameters, observed at an inclination angle of approximately 20° from the approaching jet.

## Abstract (web summary)

An imaging algorithm using closure-invariant formalism and diffusion-based reconstruction applied to supermassive black hole very long baseline radio interferometry datasets enables independent recovery of the ring-like shadow and mass that is free from the usual calibration and imaging assumptions.

## Main

The M87* supermassive black hole, with a mass of *M*_BH_ = (3–7) × 10^9^ *M*_⊙_ (refs. ^10–12^) located at a distance of *D* = 16.8 Mpc (refs. ^13,14^), offers a unique window into horizon-scale general relativistic physics. Its appearance on event horizon scales is expected, on the basis of general relativistic predictions, to be dominated by a central shadow, which, for a non-rotating Schwarzschild black hole, is characterized by a critical impact parameter that is proportional to the black hole mass^15^ as $3\sqrt{3}G{M}_{{\rm{BH}}}/({c}^{2}D)$, in which *G* is the gravitational constant and *c* is the speed of light. To observe with a spatial resolution capable of detailing the M87* black hole shadow requires unprecedented μas-scale angular resolution, roughly equivalent to the angle subtended by the width of a strand of human hair more than 1,000 km away. Achieving this feat requires a combination of millimetre wavelengths (*λ* ≈ 1 mm) and an effective aperture size comparable with the diameter of the Earth.

Such a synthesis of an Earth-sized aperture was realized through very long baseline interferometry (VLBI) techniques by correlating the signals from a global network of independent radio observatories, collectively referred to as the Event Horizon Telescope (EHT)^1,16^. As the Earth rotates, the separation vectors between pairs of geographical stations sweep out elliptical arcs that incrementally fill the Earth-sized synthetic aperture. Although this technique yields the requisite angular resolution to image the M87* shadow, the resulting virtual aperture remains substantially unfilled and lacks the sensitivity and reconstruction fidelity of a filled aperture. The data sparsity problem is further compounded by the requirement of calibrating rapid, station-based atmospheric phase and amplitude fluctuations on continental-scale separations, which often occur on timescales shorter than the coherence time of the observations.

These inherent challenges make image reconstruction from VLBI and EHT datasets an ill-posed problem, highly sensitive to instrument calibration and post-processing heuristics. Present algorithms regularly use manually tuned and independently weighted data terms and regularizations to bias the solution towards plausible morphologies, which can inadvertently steer the reconstruction towards preferred solutions. The consequences are divergent representations of the fine-scale features of M87*, such as the surface brightness distribution and hotspot localization^3,5–7,17,18^. Using traditional approaches, including CLEAN-based algorithms or hybrid mapping with self-calibration^19–23^, independent analyses showed that the resulting reconstruction is sensitive to methodological and hyperparameter selections, such as image initialization^6^ or adopted field of view^8^, with some independent analyses demonstrating reconstructions without a clear ring-like structure^8,9^, suggesting that, if the field of view extends to circular diameters beyond 120 μas, a single elongated bright spot solution is favoured over a ring^8^.

To resolve the tension around the M87* results and advance beyond methodological uncertainties, we transition from traditional approaches towards a more autonomous and objective framework. The independence of our method is primarily grounded on our use of robust interferometric observables, called closure invariants^24–27^, which capture information on source morphology that is unaffected by errors in wavefront and instrument calibration. Therefore, closure invariants constitute robust information-bearing observables in radio interferometry, underpinning high-fidelity morphology reconstructions^28–31^. Closure invariants are also insensitive to absolute position and absolute flux scale, but these are not inherent morphological parameters. In contrast to the disparate closure phases^24^ and closure amplitudes^25^ that have traditionally served as such robust observables, the closure invariants formalism^26,27^ that we use provides a unified framework that carries equivalent information while eliminating the requirement for heterogeneous treatment typically observed in the literature. By making use of the rich calibration-independent, pure morphological information carried by closure invariants, we can bypass conventional techniques that require sensitive calibration tuning and observer-driven priors.

## Interferometric imaging

To take full advantage of the closure formalism, we develop and apply the Generative Deep learning Image Reconstruction with Closure Terms (GenDIReCT)^31^ algorithm, trained on images unrelated to astronomy and designed to generatively produce data-driven image reconstructions using only closure invariants. Unlike other machine-learning-based radio interferometric imaging algorithms, our algorithm is unique in using closure invariants to condition a latent diffusion model. GenDIReCT has undergone extensive testing on both synthetic^31^ and real data^32^ and has been demonstrated to be capable of consistently recovering diverse morphological structures, without manual intervention, prescriptive priors or parameter tuning (further detail in Methods).

For the first time, to our knowledge, closure invariants are used to image M87* through our GenDIReCT algorithm, using the publicly available 1.3-mm EHT measurements from four observation epochs (5 April, 6 April, 10 April and 11 April) from 2017 (ref. ^1^), two epochs (21 April and 25 April) from 2018 (ref. ^33^) and two epochs (13 April and 18 April) from 2021 (ref. ^34^). We set several fields of view for the reconstruction (128 × 128, 225 × 225 and 450 × 450 μas^2^), in which each combination of dataset and field of view is an independent model and acts as an indirect prior on the maximum source size. For each of these models, we aggregate a large population of generative reconstructions into a single representative median image, comparing them with the Event Horizon Telescope Collaboration (EHTC) fiducial reconstruction that is reproduced using the public eht-imaging pipeline (https://github.com/eventhorizontelescope/2019-D01-02). We also derive a composite image from the pixelwise median of all reconstructions of each epoch across all fields of view aligned to the single reconstruction with the best data fidelity score.

**Fig. 1:**
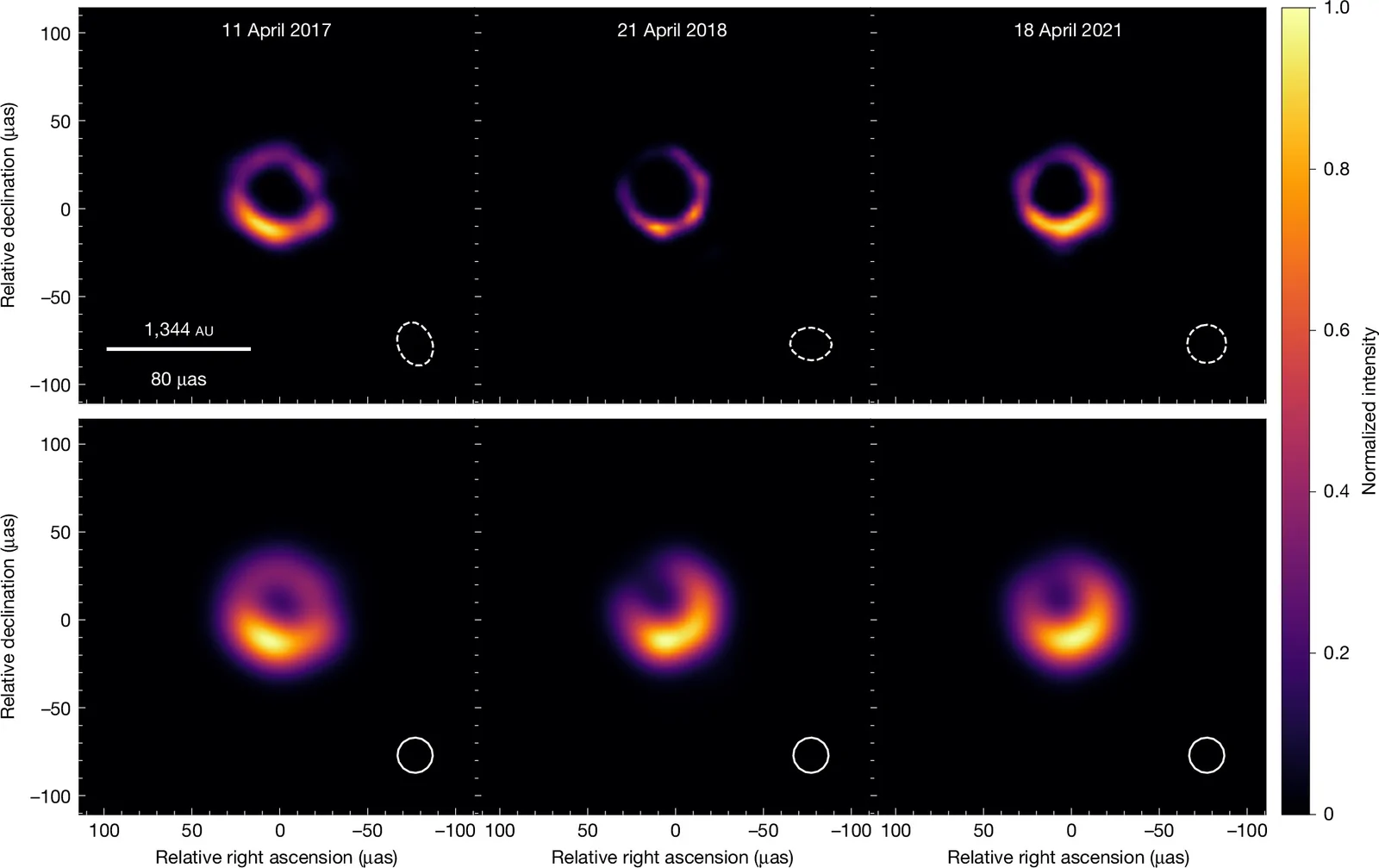
Composite image reconstructions produced with the 11 April 2017, 21 April 2018 and 18 April 2021 datasets. The dashed ellipses represent the full width at half maxima of the synthesized point spread functions. The bottom row illustrates the composite reconstructions convolved with a 20-μas circular beam, comparable with fiducial EHTC image reconstructions of M87*. Image reconstructions in the top row are unconvolved. Here intensity is illustrated in a normalized linear scale, in which the compact flux density of M87* is constrained by other means to be between 0.4 Jy and 1.0 Jy (refs. ^3,33,34^).

All datasets from independent observation epochs convincingly produce a compact, ring-like structure on an approximately 40-μas scale. On the highest-quality dataset of each year (11 April 2017, 21 April 2018 and 18 April 2021), our median reconstruction is a compact circularized ring of consistent orientation and diameter, independent of the reconstruction field-of-view window and brightened in the south or southwest direction. The appearance of the ring is most consistent with an observed inclination angle of about 20° from the approaching jet. We present the composites for our best reconstructions from these three datasets in Fig. 1.

The consistency of the source size (about 40 μas) across all fields of view is strong confirmation of a compact source. Under the most-constrained 128 × 128-μas^2^ field of view, the median reconstructed image exhibits the highest image-domain similarity score with fiducial EHTC reconstructions and the performance on data adherence metrics is competitive with those of larger fields of view, supporting the prior expectation that extended emission of the M87* jet is unconstrained by the EHT datasets^3,33^. On some infrequent reconstructions of epochs with lower-quality datasets suffering from even sparser aperture coverage^3^, lower signal-to-noise ratio or various observation deficiencies^33^, the ring-like morphology can become less clear, as noted by independent studies^8^. Consequently, the suggested sensitivity of image morphology to the assumed field of view reported in independent reanalyses and demonstrated for the 5 April 2017 dataset^8^ is possibly an artefact of insufficient aperture coverage or low-quality data. In our work, when higher-quality datasets are considered, the sensitivity to field of view disappears, contrary to predictions based on the traditional hybrid mapping approach^8^. Our work thus yields a robust, independent verification of the ring-like geometry characteristic of the M87* black hole shadow image.

In contrast to regularized maximum likelihood methods in the publicly available imaging pipelines used by the EHTC on M87*, our method does not require manually prescribing an image initialization. For instance, the eht-imaging pipeline applied on M87* is initialized with a 40-μas full width at half maximum (FWHM) Gaussian, which serves as a powerful prior on the characteristic source size. These methods typically exhibit marked sensitivity to the starting model^6^. When the eht-imaging pipeline is initialized with Gaussian priors larger than 40 μas (that is, 80–120 μas), the resulting image reconstructions exhibit phantom repetition or aliasing artefacts, which is a common issue for VLBI imaging algorithms^28,35^.

It is important, therefore, that we consistently recover a single, compact source morphology in the absence of manual prescriptions. When our model is trained on different fields of view, the model learns an implicit statistical prior from its diverse training library conditioned on the input interferometric observables, namely, closure invariants. The convergence towards a stable, singular ring structure across varying fields of view indicates that the our model is successfully inferring the intrinsic source geometry from the data itself, rather than operating within the constraints of a manually selected initialization. The autonomy and capacity for imaging directly from calibration-independent observables of GenDIReCT represents a notable step towards objective blind imaging in the challenging data-sparse VLBI regime. By unequivocally recovering the ring-like structure of M87* across several datasets through this approach, we provide a rigorous cross-validation of published EHTC results.

## Morphological analysis

We quantitatively demonstrate consistency with the EHTC image reconstructions by measuring ring characteristics from our reconstructed images and comparing them with published parameters. We apply an independent implementation of the image-domain feature extraction method used by the EHTC^3^ to measure ring properties from reconstructed event-horizon-scale images, including ring diameter, width, orientation (measured anticlockwise from north), azimuthal asymmetry and central brightness fraction. The measured properties from our median reconstructions of all datasets are presented in Table 1. Each cell represents the mean parameter measured from independent reconstructions on all three fields of view.

**Table 1:** Tabulated ring properties measured from image-domain feature extraction

Date	Ring diameter, ${\bf{2}}{\bar{{\boldsymbol{r}}}}_{{\bf{pk}}}$ (μas)	Ring width, *w*_r_ (μas)	Orientation, *η* (°)	Azimuthal asymmetry, *A*	Central brightness fraction, *f*_c_
5 April 2017	42.0 ± 3.6	17 ± 2	143 ± 6	0.29 ± 0.04	0.115 ± 0.062
6 April 2017	43.1 ± 2.3	14 ± 2	148 ± 11	0.33 ± 0.04	0.032 ± 0.026
10 April 2017	43.3 ± 0.4	22 ± 7	177 ± 7	0.36 ± 0.07	0.135 ± 0.066
11 April 2017	43.3 ± 1.0	16 ± 3	167 ± 6	0.20 ± 0.01	0.037 ± 0.028
21 April 2018	42.7 ± 0.5	11 ± 1	205 ± 10	0.31 ± 0.01	0.004 ± 0.004
25 April 2018	43.3 ± 4.6	36 ± 13	206 ± 2	0.41 ± 0.18	0.270 ± 0.135
13 April 2021	40.4 ± 1.7	17 ± 9	191 ± 2	0.43 ± 0.03	0.002 ± 0.001
18 April 2021	39.9 ± 1.8	12 ± 1	197 ± 4	0.26 ± 0.01	0.004 ± 0.002
Mean	42.2 ± 1.5	–	–	–	–

Each cell represents the mean and standard deviation estimate from our unconvolved reconstructions on all three fields of view. The mean and standard deviation across all epochs for the ring diameter is listed as a summary statistic.

**Fig. 2:**
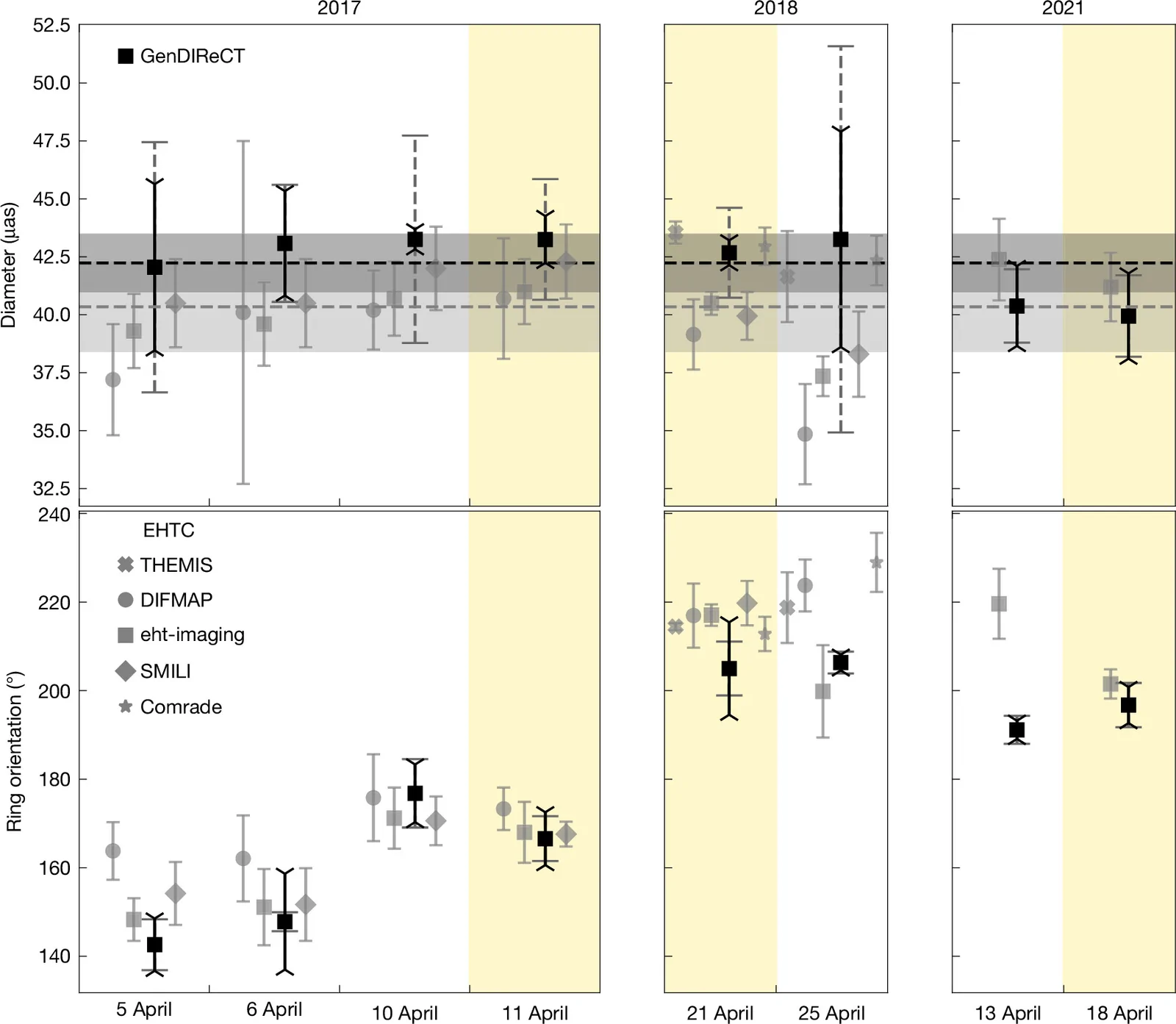
Measured ring diameter and orientation. Comparison of ring diameter and orientation measured from image-domain feature extraction applied on our reconstructions with values from the EHTC, separated by observation epoch. EHTC measurements on each epoch correspond to different imaging algorithms (from left to right: THEMIS, DIFMAP, eht-imaging, SMILI and Comrade). Our measurements are presented with two estimates of uncertainty: the dashed line delineates the dispersion (along the azimuthal axis for diameter and along the radial axis for orientation) in the image-domain feature extraction method and the solid line with triad cap indicates the standard deviation across fields of view, which is unique to our method. For the diameter, we illustrate the mean and 1*σ* standard deviation for EHTC methods and our method with the dashed line and sheath. The highest-quality dataset of each year is highlighted in yellow.

The measured ring orientation and diameter remain relatively stable between the GenDIReCT and EHTC imaging methods. In Fig. 2, we compare our image-domain measurements derived from our reconstructions with the EHTC analogue. There is a clear anticlockwise trend in the ring orientation angle as measured by all methods between the 5 and 6 April reconstructions with 10 and 11 April in 2017, as well as between the 2017 and 2018/2021 observations. However, the evolution in 2017 may be caused by uncertainty in the azimuthal structure brought about by the sparse aperture coverage of observations taken on 5 and 6 April. Nevertheless, the evolution in ring orientation between the 2017 and 2018 observations is noteworthy, attributable to the turbulent nature of black hole accretion^36^. In particular, the observed ring orientation in 2018 and 2021 are closer in alignment with the large-scale forward jet of M87* compared with that in 2017.

Conversely, the measured ring width and central brightness fraction are heavily influenced by methodological differences, as seen in the variation between imaging algorithms. For instance, reconstructions from the DIFMAP algorithm^37^ are convolved with a 20-μas circular beam, which inflates the ring width compared with regularized maximum likelihood methods and our algorithm. Likewise, the azimuthal asymmetry parameter is unstable between different days and different methods, which is indicative of sensitivity to variation in the aperture coverage. Therefore, the ring width and central brightness fraction probably do not reflect intrinsic source properties.

**Fig. 3:**
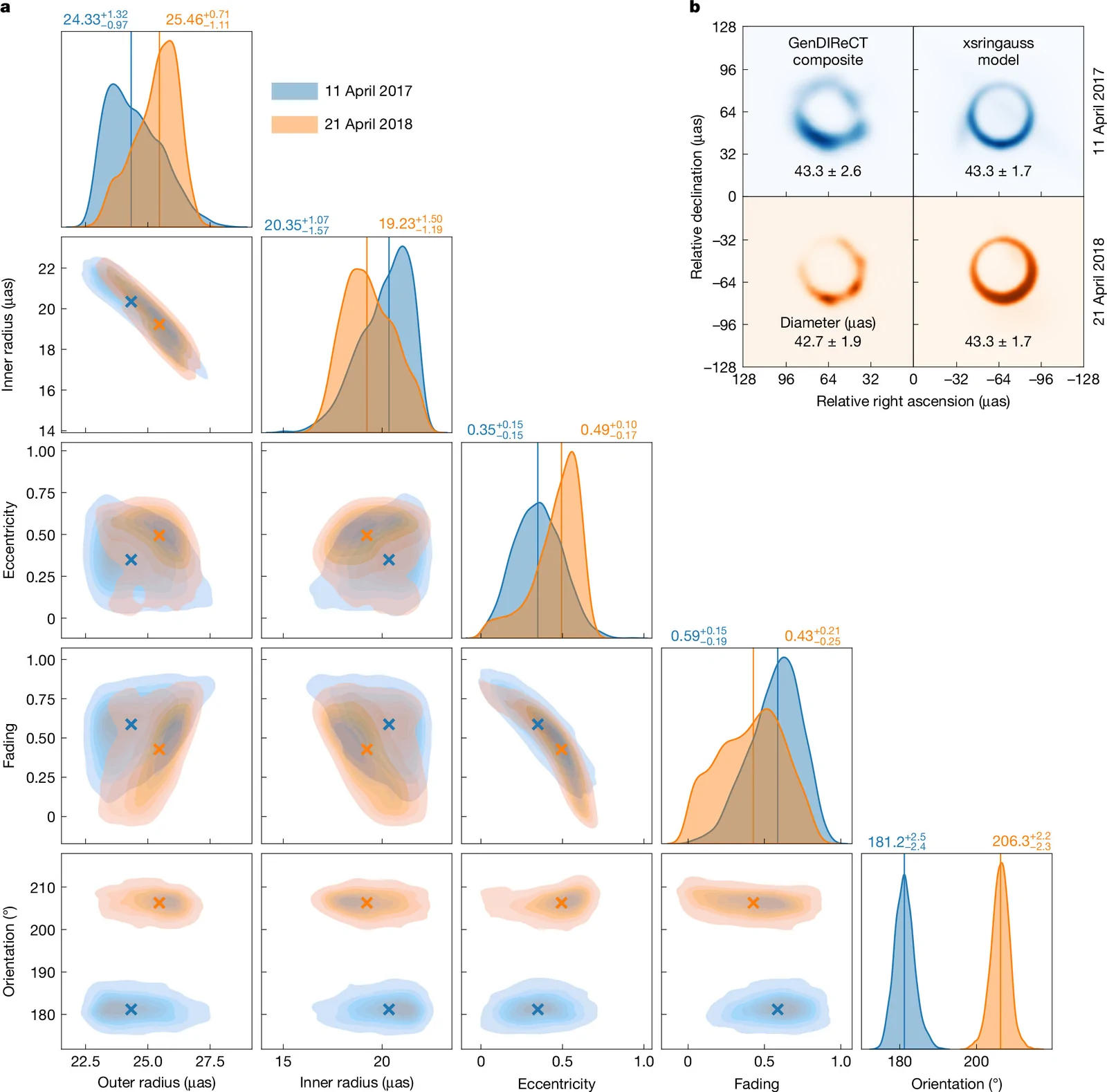
Posterior covariance from xsringauss geometric modelling on the 11 April 2017 and 21 April 2018 datasets. **a**, The one-dimensional and two-dimensional marginalized posterior distributions and median values for the outer radius, inner radius, eccentricity, fading parameter and orientation. **b**, The composite images created from all GenDIReCT reconstructions across all fields of view are presented in comparison with the fitted median xsringauss sky models. The diameter is measured with image-domain feature extraction and the corresponding uncertainty is from azimuthal dispersion rather than the sample variance across several fields of view.

To further cross-validate the measured ring parameters, we use a complementary Bayesian forward-modelling framework to fit the observed closure invariants using a superposition of primitive geometric shapes with analytic visibilities. With the ring-like structure firmly established by our independent, calibration-insensitive and non-parametric imaging algorithm, we proceed to construct our geometric composite using the xsringauss^4^ model as a foundation. We include one centralized large Gaussian and two elliptical nuisance Gaussians with independent spatial offsets to help capture non-ring short-spacing flux and extraneous emission. Using the DYNESTY nested sampling algorithm^38^, we perform parameter estimation for the xsringauss model for each epoch. Figure 3 shows the posterior covariance from geometric modelling of the 11 April 2017 and 21 April 2018 datasets, in which the one-dimensional and two-dimensional marginalized posterior distributions of the outer radius, inner radius, eccentricity, fading parameter and orientation are illustrated. We apply the image-domain feature extraction method on the geometric image model derived from the median value of each one-dimensional posterior distribution to measure all characteristic ring parameters. Posterior covariances for all other datasets are provided in Methods. In all epochs, we measure a mean diameter and standard deviation of *d* = 42.3 ± 1.1 μas from forward geometric modelling of closure invariants, which is compatible with the mean *d* = 42.2 ± 1.5 μas from our non-parametric image reconstructions.

Inferring the gravitational radius of the black hole, and hence its mass, from the measured ring diameter requires independently calibrating the imaging method with synthetic data from simulated models of lensed black hole accretion emission^4^. The prior expectation for the mass of the central black hole in M87 is primarily sourced from high-quality gas^12^ and stellar^11^ dynamical estimates, which measured ${M}_{{\rm{gas}}}=3.{5}_{-0.7}^{+0.9}\times 1{0}^{9}{M}_{\odot }$ and *M*_star_ = (6.6 ± 0.4) × 10^9^ *M*_⊙_, respectively. Using our measured ring diameter, distance measure of $16.{8}_{-0.7}^{+0.8}\,{\rm{Mpc}}$ and nominal scaling parameters for the gravitational radius, we estimate a black hole mass of *M*_BH_ = (6.5 ± 0.5) × 10^9^ *M*_⊙_ for M87*.

Our independently measured ring diameters and inferred black hole mass from GenDIReCT imaging and geometric modelling are therefore in excellent agreement with previous stellar dynamical mass measurements^11^ and with image-domain feature extraction on the imaging methods applied by the EHTC. By contrast, the smaller ring diameter predicted by the black hole mass estimate from gas dynamics would have been challenging to resolve given the EHT beam dimensions of approximately 20 μas. Future observations with the James Webb Space Telescope Near-Infrared Spectrograph integral field unit will provide further detail on the kinematics within the central arcsecond of the M87 core^39^, producing finer constraints on the M87* black hole mass.

**Fig. 4:**
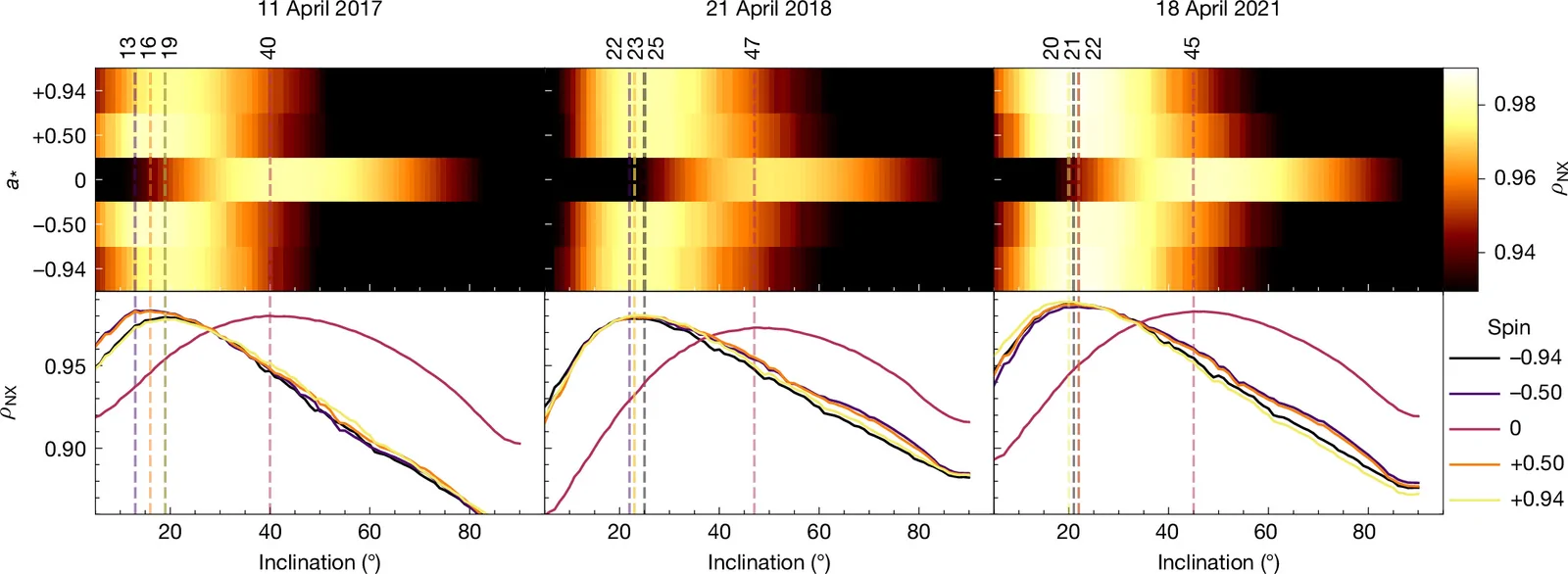
Comparative image-domain inclination analysis. Image-domain similarity score across the range of black hole spin and observed inclinations for simulated M87* images compared with composite reconstructions for the highest-quality datasets of each year. Top, illustration of the covariance in image-domain similarity with black hole spin and inclination. Bottom, relationship between inclination and image-domain similarity, separated by black hole spin.

The appearance of the black hole shadow is also sensitive to the black hole spin, *a*⁎, and observed inclination angle. We compare our composite M87* reconstructions with synthetic 230-GHz images of M87* produced by the IPOLE algorithm^40^, which integrates the covariant radiative transfer equation over relativistic photon trajectories across fluid data from general relativistic magnetohydrodynamic (GRMHD) simulations^41^. Quantitative analysis using the normalized cross-correlation similarity score, *ρ*_NX_, comparing beam-convolved synthetic images with image reconstructions, identifies a clear sensitivity to inclination. We present the results based on composites from highest-quality datasets for all years in Fig. 4. Note that we do not explore inclination angles >90°, as the resulting synthetic images are comparable with an inversion of the spin direction. Although the highest similarity scores favour a non-zero black hole spin, our analysis does not constrain the spin magnitude, consistent with previous analyses with EHT^36^ and non-EHT data^42^. Altogether, the best-scoring inclination is approximately 20° and, although the best-scoring inclination in the *a*⁎ = 0 case is much higher, such models are disfavoured a priori by independent considerations of jet power^43–45^ and precession^42^. Consequently, the results from our image-domain comparative analysis are generally consistent with the fiducial 17.2° ± 3.3° jet viewing angle inferred from 43-GHz VLBI observations^46,47^. Future horizon-scale imaging with space-based VLBI offers the potential to resolve the M87* photon ring^48^, which would enable a direct and precise determination of the black hole spin, at present unconstrained by an image-domain analysis.

## Methods

### EHT datasets on M87*

This work uses the publicly available network-calibrated datasets of M87* (https://eventhorizontelescope.org/for-astronomers/data), reduced through the EHT-HOPS pipeline, on the basis of observations obtained in 2017 (ref. ^2^), 2018 (ref. ^33^) and 2021 (ref. ^34^). In 2017, EHT observations of M87* were conducted with seven separate facilities^1^: the Atacama Large Millimeter/submillimeter Array (ALMA)^49,50^, the Atacama Pathfinder Experiment telescope (APEX)^51^, the Large Millimeter Telescope (LMT)^52^, the Pico Veleta IRAM 30-m telescope (PV)^53^, the Submillimeter Telescope Observatory (SMT)^54^, the James Clerk Maxwell Telescope (JCMT)^55^ and the Submillimeter Array (SMA)^56^, which heterogeneously and sparsely sample the Fourier plane as the Earth rotates. Furthermore, the Greenland Telescope (GLT)^57,58^ participated in the 2018 observations for the first time as part of the EHT array, which improved the aperture coverage, particularly along the north–south direction. For 2021, the 12-m Kitt Peak (KP)^59^ telescope and the upgraded Northern Extended Millimeter Array (NOEMA)^60^ facility joined EHT observations for the first time.

The 2017 observations of M87* are spread over four separate epochs (5, 6, 10 and 11 April), whereas observations in 2018 and 2021 are separated into two epochs each (21/25 April for 2018 and 13/18 April for 2021). The datasets with the best single-day aperture coverage each year are 11 April, 21 April and 18 April, which we use as our fiducial datasets, whereas datasets on other days are used as cross-validation. We consider the data taken over two approximately 2-GHz bands centred at 227.1 (low) and 229.1 GHz (high), which we merge into a single dataset.

The interferometric data on M87* comprise complex visibilities, correlated from signals between pairs of stations, referred to as a baseline, which are, by the van Cittert–Zernike theorem^61^, related to the source brightness distribution by a two-dimensional Fourier transform after certain approximations^62^. Visibilities can be sequenced into scans, on the basis of the time difference between observations, from which we obtain coherently averaged visibilities. In this work, closure invariants are generally computed from scan-averaged visibilities. With typical scan lengths on the order of five minutes, temporal averaging can potentially introduce residuals from time-dependent gain fluctuations to closure invariants. We have internally validated the robustness of our imaging products by computing closure invariants at the smallest possible cadence of the science-ready dataset (typically 10 s) before scan-level averaging. Following the procedure in the publicly available M87* imaging pipeline (https://github.com/eventhorizontelescope/2019-D01-02), we rescale short baselines to the total flux density estimated from the ALMA and APEX baseline to excise the contribution from extended flux. We have also internally validated that the short baseline rescaling procedure does not affect the recovered morphology from our imaging algorithm. For quantitative comparison, we generate fiducial images from the publicly available eht-imaging pipeline to represent EHTC reconstructions.

### Closure invariants

Closure invariants are calibration-independent interferometric measurables produced from a generalized formalism of traditional closure phase^24,63^ and amplitudes^25^, with both a co-polar version^26^ and a polarimetric version^27^. GenDIReCT uses co-polar closure invariants constructed from elementary triangular plaquette variables called ‘advariants’. The complete minimal set of independent closed triads can be constructed from an array of *n* stations by selecting all pairs of array elements (*a*, *b*) pinned on a fixed reference vertex indexed at 0,

1 $${{\mathcal{A}}}_{0{ab}}^{{\prime} }={{\mathcal{V}}}_{0a}^{{\prime} }({{\mathcal{V}}}_{{ab}}^{{\prime} \ast }{)}^{-1}{{\mathcal{V}}}_{b0}^{{\prime} }=|{g}_{0}{|}^{2}{{\mathcal{A}}}_{0{ab}},$$

in which ${{\mathcal{V}}}_{{ab}}^{{\prime} }$ are corrupted visibilities measured from the baseline between stations *a* and *b* and |*g*_0_|^2^ is the unknown scaling factor associated with the reference station, which is shared by all complex advariants constructed in this way. By normalizing all advariants by the *L*_2_-norm, for example, we produce ${N}_{{\rm{s}}}^{2}-3{N}_{{\rm{s}}}+1$ real-valued independent closure invariants, whose form is different but carry information equivalent to that in traditional closure amplitudes and phases. In the co-polar formalism of closure invariants, traditional closure phases can be identified trivially from the phases of the advariants, whereas closure amplitudes can be derived from specific pairs of advariants. A key advantage of this formalism over traditional closure amplitudes and phases is that it avoids the heterogeneous treatment that phases and amplitudes necessitate. In internal validation tests, closure invariants as the sole likelihood term was found to be preferable over the independently weighted (log-)closure amplitudes and closure phases in terms of convergence and data efficiency.

Various selections of the reference station can affect covariances between closure terms, despite carrying equivalent information. A different selection of reference station can influence the signal-to-noise ratio in the derived closure invariants but does not greatly influence the resulting image reconstruction^32^. For this work, to avoid bias from a single reference station selection, we present the combined results from two GenDIReCT models for each dataset, trained consistently on the first and the second most sensitive stations as the reference stations in each scan. Selecting from among the most sensitive stations maximizes closure-invariant signal-to-noise ratio and minimizes covariances between closure terms^64^.

### GenDIReCT imaging

The GenDIReCT^31^ algorithm is an imaging algorithm that uses co-polar closure invariants^26^ for calibration-independent imaging under the constraints of sparse aperture coverage characteristic of VLBI data. The architecture comprises a latent diffusion model^65^ conditioned on closure invariants and a multilayer convolutional neural network (CNN) used to optimize the closure invariants likelihood. Traditional imaging algorithms, such as hybrid mapping and self-calibration followed by deconvolution, begin with a simple initial model and iteratively solve for antenna-based complex gains and the image model to converge on a final solution that matches the measured data. These methods can be sensitive to and biased by image initialization and calibration errors, particularly in sparse and low signal-to-noise measurements^6,19,22^. Our algorithm bypasses the limitations of conventional techniques by using closure invariants as the robust interferometric measurable and produces an image reconstruction directly in a single forward pass. Furthermore, in contrast to the newer regularized maximum likelihood methods that can also image using closure invariants as the sole data term^28,35,66,67^, our algorithm provides a generative solution that does not require a user-specified, hand-tuned image initialization at inference time. Other machine learning interferometric imaging techniques have been presented in the literature, on the basis of CNNs^68–72^, normalizing flow^73,74^, denoising diffusion^74,75^, classifiers^29,76^, adversarial networks^76,77^ and transformers^30^. Although there are some similarities with other diffusion-based imaging algorithms^74,75^, GenDIReCT is unique in its closure-conditioned latent diffusion model and, notably, in its use of homogeneous closure invariants rather than the traditional closure phases and closure amplitudes that require heterogeneous treatment. In this section, we provide a brief overview of our algorithm and its past applications.

The neural network architecture is composed of two distinct components. The first component is a conditional U-Net^78^ based on textually conditioned image generation architectures^65^ with denoising diffusion probabilistic models^79^, which has emerged as a highly effective image generation technique, capable of modelling complex data distributions. Our implementation is a latent diffusion model consisting of three convolutional layers set in a symmetric contracting and expanding pathway with residual connections^80^ from the contracting path that map features to their mirrored expanding layers. In the contracting path, we use one downsampling layer, followed by two downsampling layers with cross-attention, in which the closure-invariant features adjust convolutional features through the cross-attention mechanism, which conditions the denoising process. On the upsampling path, we implement two upsampling layers with self-attention and one final upsampling layer without attention. By having no cross-attention layers in the upsampling path, the result is a robust conditional denoising network that performs well even with noise from real observations. The latent shape is 4 channels of 16 × 16 grids, which is downsampled from the original 64 × 64 total intensity image grid using a pretrained autoencoder^30^. The second component of the GenDIReCT architecture is a standard multilayer CNN composed of three sequential downsampling layers, designed to compress a large set of diffusion-sampled images (each considered a separate channel) into a representative data-consistent single-channel image reconstruction. We note that we have experimented with several design variations and found this version to be suitable in all tested cases, but better-performing alternatives for specific datasets may be possible.

GenDIReCT works by learning realistic image characteristics (such as positivity and smooth brightness distribution) from a non-astronomical image training dataset, primarily composed of augmented CIFAR-10 natural images^81^. The conditional latent diffusion model generates sensible image priors, which the multilayer CNN uses as input to optimize the data likelihood and refine the final image reconstruction. As a generative imaging algorithm, our algorithm has the potential to handle degeneracies and visualize multimodalities in the solution space, although they should not be interpreted as image reconstruction uncertainty under a Bayesian framework. Generally, appearance of minor artefacts in low-sensitivity regions, different levels of smoothness and sharpness or super-resolution variations are acceptable consequences of a diversity in potential solutions to the ill-posed image reconstruction problem. However, methodological and hyperparameter sensitivity, or inability to satisfy the underlying measurements, constitute signs that a reconstruction is not robust. By training on unconvolved ground-truth models, GenDIReCT achieves super-resolved reconstructions and enables generative visualization of structural variations that remain strictly consistent with the observed data. Accordingly, unless otherwise indicated, we present unconvolved GenDIReCT outputs optimized to be consistent with the measurements.

We have extensively tested our algorithm on synthetic datasets with the EHT or next-generation EHT (ngEHT) array^31^, as well as non-horizon-scale EHT datasets on active galactic nuclei, 3C 279 and Centaurus A (ref. ^32^). On synthetic datasets, our algorithm generalizes well to untrained morphologies and maintains >90% image similarity with ground-truth images at signal-to-noise levels well below the datasets of M87*. Moreover, its performance on quantitative metrics is comparable with other state-of-the-art imaging algorithms on the ngEHT total intensity analysis challenge^82^. For the EHT dataset on 3C 279, our reconstruction is 98% similar to the EHTC ref. ^83^, reproducing the core-jet morphology across several independent epochs and enabling direct measurement of the superluminal relative proper motion between components in the image domain. For the EHT dataset on Centaurus A, the EHTC ref. ^84^ is reproduced with 91% similarity with statistically compatible brightness ratios and opening angle between the north and south edge-brightened jet ridges. These extensive tests on real and synthetic data demonstrate that our algorithm is capable of consistently recovering diverse morphological structures, without manual intervention or prescriptive priors.

Before applying to the real data, each trained model is extensively tested on an array of internal validation tests, which include quantifying its performance degradation with out-of-distribution image morphologies and varying levels of data corruption, accounting for the heterogeneous sensitivity of the array^32^. Only after passing internal validation are the models unblinded on real data to minimize risk of confirmation bias. No alterations are made to the model between the validation procedure and the unblinding, or post-unblinding.

**Extended Data Fig. 1:**
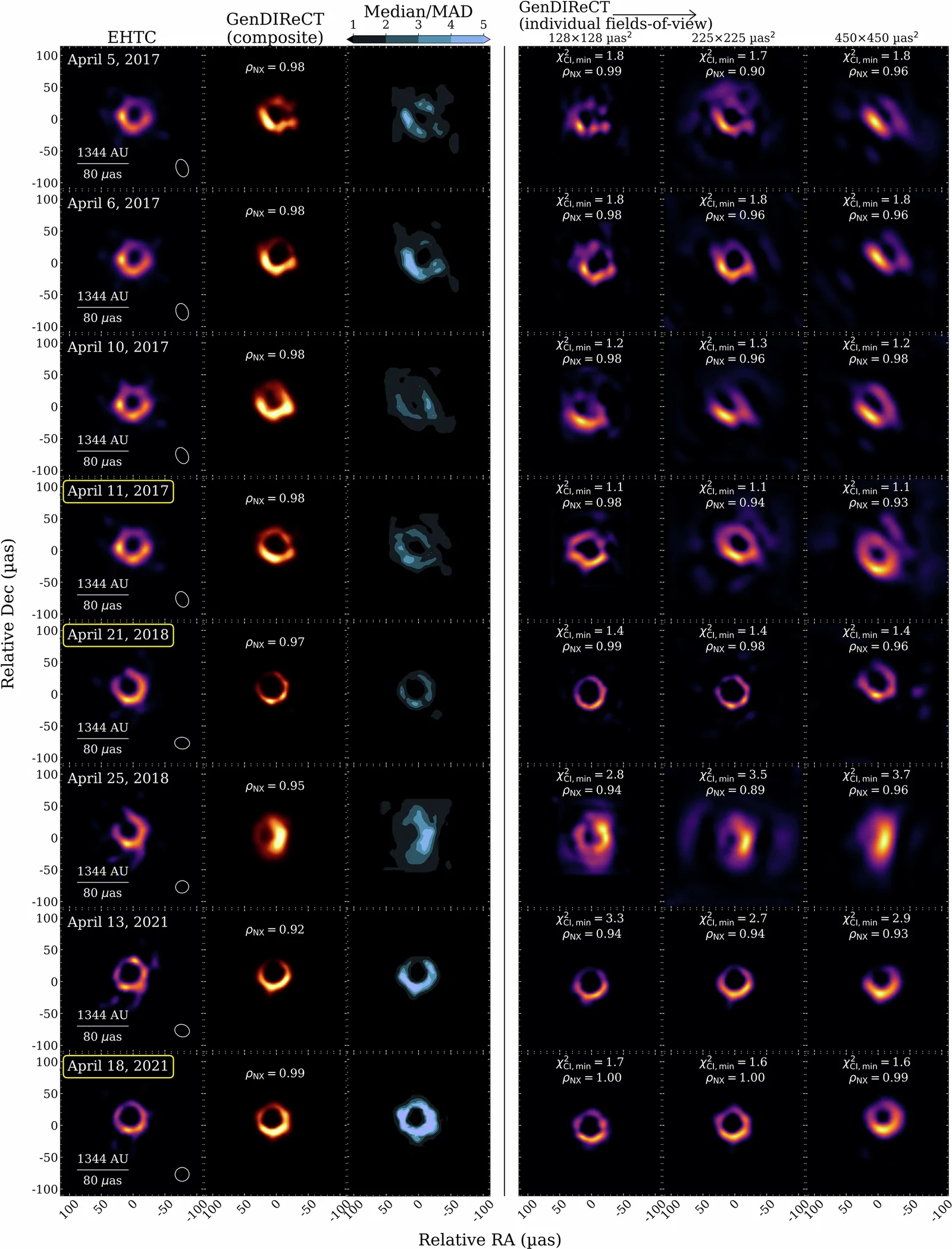
Individual dataset composites and reconstructions across multiple fields-of-view compared to EHTC references. Left panels, fiducial reconstructions from eht-imaging for the single-epoch datasets. The highest-quality dataset of each year is highlighted with a gold border. Right panels, corresponding composite GenDIReCT reconstruction, median-to-MAD ratio and median representative reconstructions across several fields of view. Data fidelity (${\chi }_{{\rm{CI}},\mathrm{min}}^{2}$) on median reconstructions and image similarity (*ρ*_NX_) scores are illustrated, for which *ρ*_NX_ is measured relative to the EHTC fiducial image reconstruction. All panels are shown on a 225 × 225-μas^2^ window.

For this work, we train our machine learning architecture with synthetic data derived using the data coverage from all four 2017 and both 2018/2021 observing days independently, combining the two 2-GHz bands into a single scan-averaged dataset for each epoch. We choose 128 × 128-μas^2^, 225 × 225-μas^2^ and 450 × 450-μas^2^ reconstruction fields, each on 64 × 64-pixel grids as predetermined by the architecture. Generally, we represent the aggregate reconstruction for each dataset by the median of all aligned reconstructions. We find the median representation to be more robust against outliers or source alignment issues and a better representation of the bulk of individual image reconstructions. The aggregate reconstructions for all datasets and fields of view are shown in Extended Data Fig. 1 alongside EHTC comparison reconstructions, GenDIReCT composites and a median to median absolute deviation (MAD) ratio panel. The median-to-MAD ratio is presented as a visualization of GenDIReCT’s reconstruction confidence^31^, in which features of low variance relative to the median pixel intensity (that is, high reconstruction confidence) are highlighted with filled contours.

**Extended Data Fig. 2:**
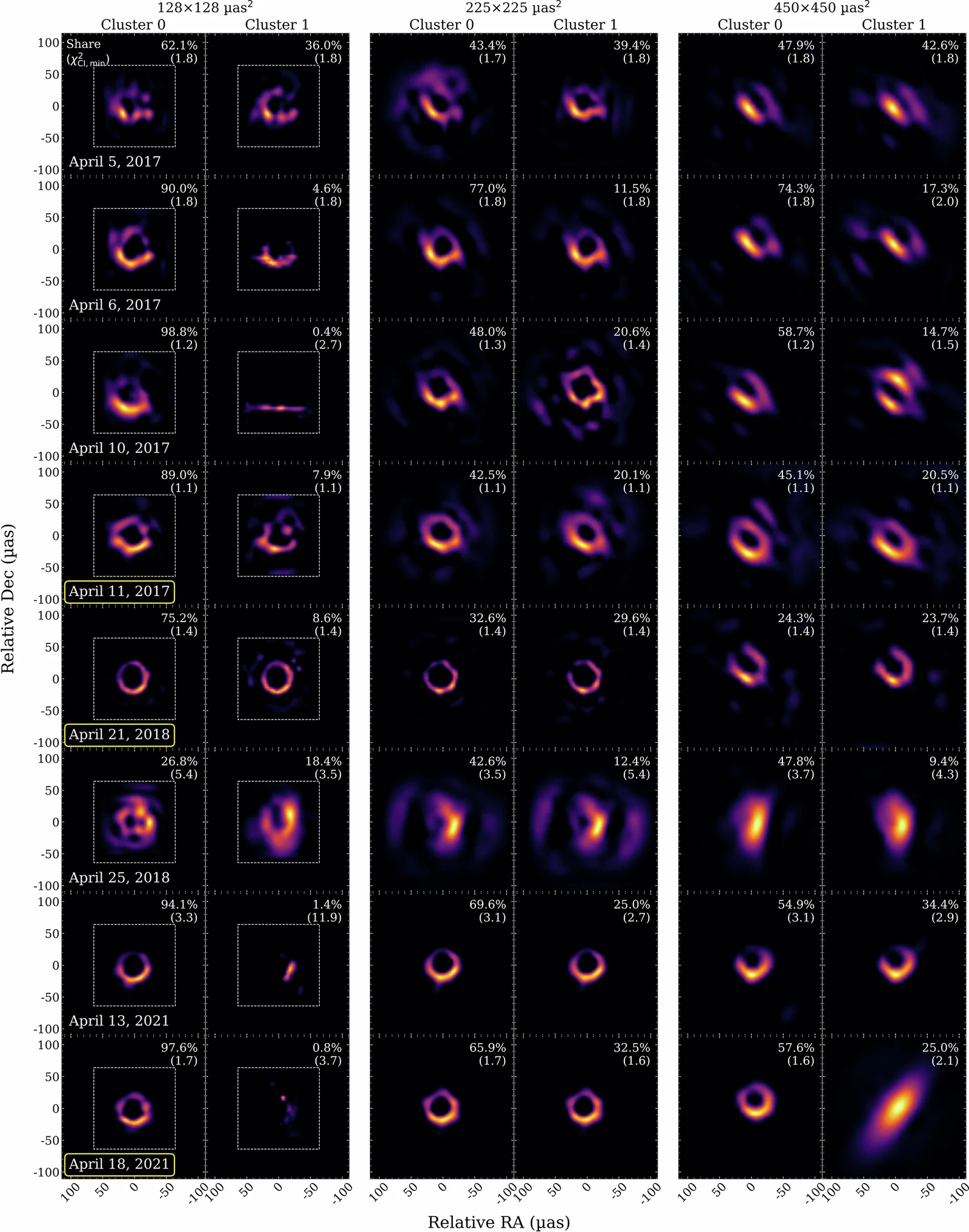
Clusters of GenDIReCT reconstructions across 128 × 128, 225 × 225 and 450 × 450-μas^2^ fields of view. The two most-populated clusters for each field of view are presented in descending order from left to right. Each row represents a separate single-epoch dataset and the highest-quality dataset of each year is highlighted with a gold border. The population of each cluster is represented by a percentage fraction alongside the minimum ${\chi }_{{\rm{CI}}}^{2}$ in parentheses. Images containing the five most-populated clusters can be found in the GenDIReCT repository.

One of the advantages of the generative imaging process of our algorithm is the potential to explore different imaging solution modes. We consider all observation epochs and all fields of view, presenting the median reconstructions from the most populated solution clusters in Extended Data Fig. 2. Image reconstructions are clustered by Hamming distance in their perceptual hash representation^32^ to illustrate multimodal solutions. Each cluster is also presented with the best data fidelity score in parentheses. Often, more than 50% of all reconstructions reside within the top two most populous and perceptually similar clusters. This clustering behaviour justifies the pixelwise median as a statistically sound and accurate aggregate representation. The M87* clustering results clearly demonstrate that the differences between reconstruction clusters for the observation days with the highest-quality data are in the surface brightness and extent of extraneous amorphous emission surrounding the ring, rather than fundamental changes to the ring structure itself. For other datasets, solution clusters that are not as clearly ring-like are typically of sparse occurrence in comparison with the bulk of reconstructions and exhibit a lower data fidelity score. On all fields of view, there is no non-ring solution that is favoured over ring-like morphology in the share of the reconstructed image population or data fidelity score. The most ambiguous case is that of the 25 April 2018 dataset, which is missing data from the LMT, resulting in reduced image fidelity compared with the 21 April 2018 observations. The absence of LMT-related baselines created a substantial gap in *uv* coverage at spatial frequencies corresponding to about 1 Gλ, as the LMT–SMT baseline provided the unique long-baseline constraint for structure in this regime^33^.

**Extended Data Fig. 3:**
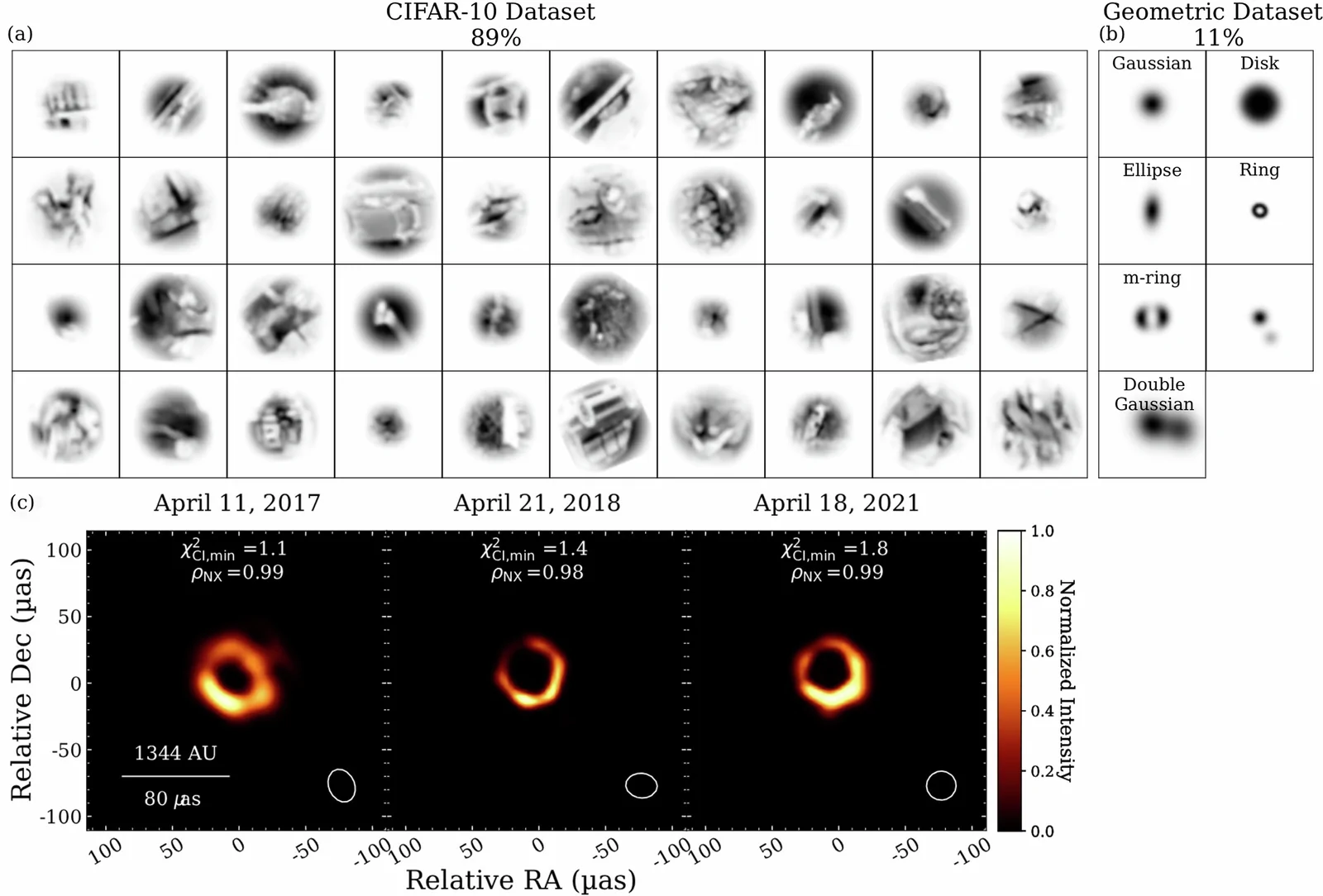
Example grid of augmented image morphologies used for training GenDIReCT and reconstructions in the absence of the geometric dataset. **a**, Image grid showing an assortment of augmented non-astronomical CIFAR-10 images that constitute most of the training dataset. **b**, Panels illustrate classes of simple geometric shapes used in training, which are not required to produce robust image reconstructions of M87*. Percentages indicate the fraction of the total training dataset composed by each class. **c**, Alternative GenDIReCT composites for the highest-quality single-day datasets from observations in 2017, 2018 and 2021, without geometric shapes in the training data. The image similarity score is with reference to the EHTC fiducial reconstructions produced with eht-imaging.

Unless otherwise stated, all models described in this work are trained on augmented non-astronomical images from the CIFAR-10 dataset^81^, as well as simple geometric shapes, such as Gaussians, disks, rings, ellipses and *m*-rings^85^ of the first and second orders^31^. We present an example grid of augmented training images in Extended Data Fig. 3. Once trained, we run the imaging sequence for 96 total GPU hours on a single NVIDIA H100 GPU for each dataset, producing several hundred (about 300) individual image reconstructions, each drawing from 4,096 diffusion samples. The exact number of final image reconstructions depends on data volume. Although our algorithm has been shown to be generalizable to arbitrary source morphologies^31^, there is a concern that the presence of crescents and rings in the training dataset may be a source of bias for the image reconstruction. Therefore, we also investigate the outcome of training our architecture without geometric shapes in the training dataset and find no notable difference in the image reconstruction (Extended Data Fig. 3).

### Metrics

We use the reduced ${\chi }_{{\rm{CI}}}^{2}$ metric to evaluate the consistency between our image reconstructions with the measurements. The reduced ${\chi }_{{\rm{CI}}}^{2}$ metric is defined as the sum of error-normalized residuals divided by the number of independent data terms,

2 $${\chi }_{{\rm{CI}}}^{2}=\frac{1}{{N}_{{\rm{ci}}}}\mathop{\sum }\limits_{i}^{{N}_{{\rm{ci}}}}\left[\frac{{({\mathcal{C}}{(A)}_{i}-{C}_{i})}^{2}}{{\sigma }_{i}^{2}}\right],$$

in which ${\mathcal{C}}$ is an operator that maps the reconstructed image to its closure invariants, indexed by *i*, for a fixed observation arrangement and *C*_*i*_ is the corresponding closure invariant of the measurement. The total number of closure invariants is denoted by *N*_ci_ and ${\sigma }_{i}^{2}$ is the estimated uncertainty on the *i*th closure invariant. We also define the normalized residual of any data point, *i*, as $({\mathcal{C}}{(A)}_{i}-{C}_{i})/{\sigma }_{i}$.

**Extended Data Fig. 4:**
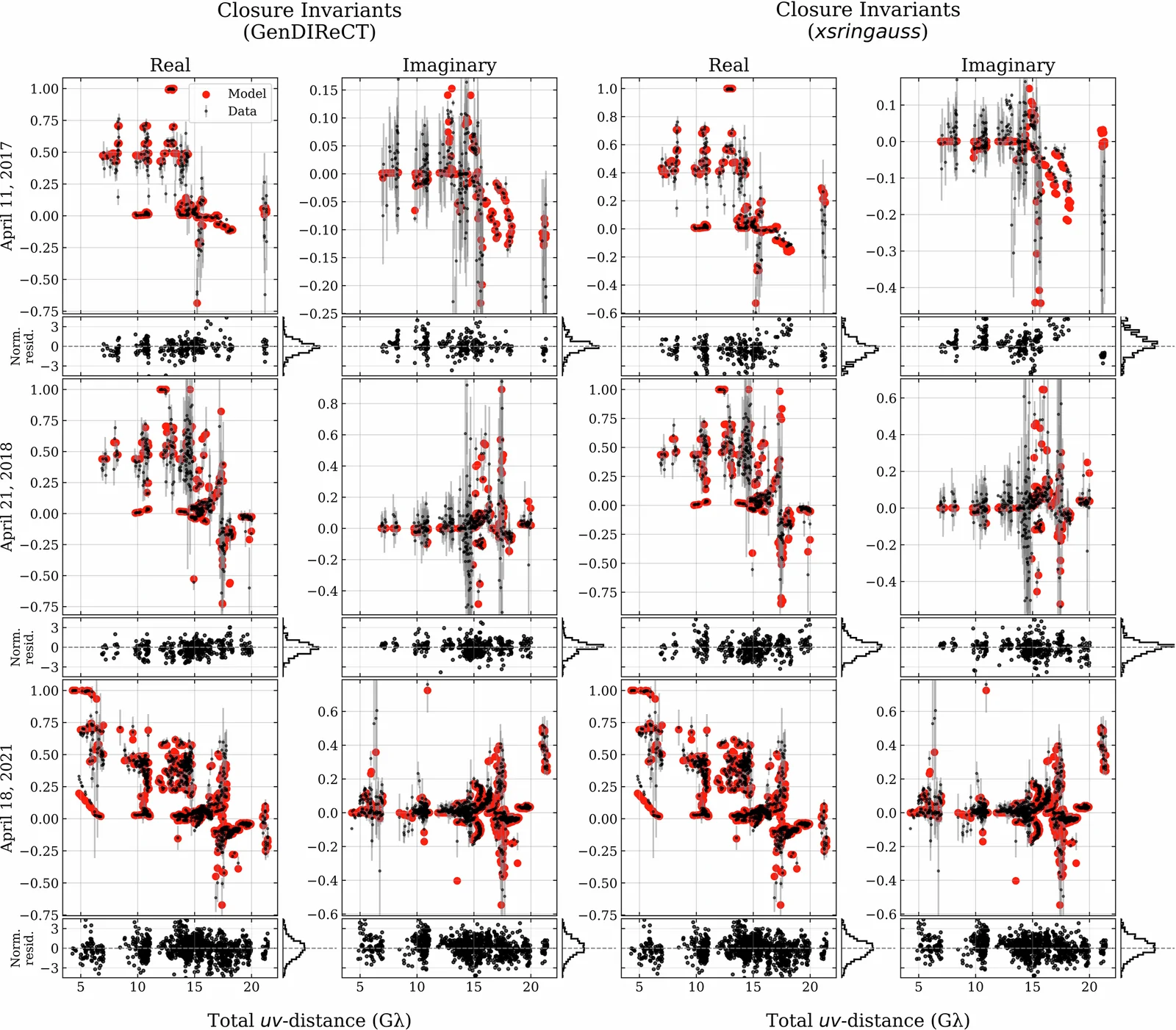
Data fidelity from the best reconstructions on each dataset. Determined by the minimum ${\chi }_{{\rm{CI}}}^{2}$ of all GenDIReCT (left) and xsringauss geometric modelling (right) reconstructions for each respective dataset, in which closure invariants are presented as real and imaginary values across the summed *uv* distance in G*λ* between all three participating stations. The residuals normalized by the expected uncertainty are presented on separate adjoining panels.

Although propagating the estimated uncertainty from each radio station’s system equivalent flux density is straightforward, the total error on each closure invariant is affected by other sources of systematic errors, including time-dependent errors from long coherent visibility averaging and limitations of the linear error propagation model. Generally, the fractional systematic uncertainty budget of 3C 279 and M87 data is about 5%, which is similar in magnitude as measured inter-pipeline differences^2^. However, in this work, we present our results without further systematic error tolerance and we consistently use as reference the highest-quality single-day EHT-HOPS-reduced pre-scan-averaged dataset of each observation year (11 April for 2017, 21 April for 2018, 18 April for 2021) when evaluating the reduced ${\chi }_{{\rm{CI}}}^{2}$ to ensure a consistent metric across reconstructions. For these datasets, we illustrate the data fidelity from the best GenDIReCT reconstruction and the xsringauss geometric model in Extended Data Fig. 4. A near-unity reduced ${\chi }_{{\rm{CI}}}^{2}$ value confirms that a reconstruction is self-consistent with the measured closure invariants but does not guarantee that the recovered morphology is correct in an absolute sense, making the reduced ${\chi }_{{\rm{CI}}}^{2}$ a necessary but insufficient metric for an accurate reconstruction.

In some cases, it is useful to measure the similarity between reconstructions in the image domain, especially when comparing the GenDIReCT reconstructions with the results from the EHTC imaging pipelines. We measure the image-to-image correspondence with the maximum normalized cross-correlation metric, *ρ*_NX_, which has the advantage of being independent of image translation and total flux scaling. The metric has been widely used to quantitatively measure image similarity, particularly with image reconstructions produced using only closure data terms^28,31,32,82^. The *ρ*_NX_ metric is defined as

3 $${\rho }_{{\rm{NX}}}({I}_{1},{I}_{2})=\frac{1}{M}\mathrm{max}|{{\mathcal{F}}}^{-1}\{{\mathcal{F}}\{\hat{{I}_{1}}\}{\mathcal{F}}{\{\hat{{I}_{2}}\}}^{\ast }\}|,$$

in which *I*_1_ and *I*_2_ are two images being compared and the hat operator, $\hat{I}=(I-\bar{I})/{\sigma }_{I}$, normalizes each image *I* by its respective flux mean and standard deviation. We denote the forward and inverse Fourier transform as ${\mathcal{F}}$ and ${{\mathcal{F}}}^{-1}$, respectively. The maximum operator ensures that the metric is independent of image translation and it is also useful for aligning separate image reconstructions. To minimize the negative influence of high-frequency artefacts on the maximum normalized cross-correlation, we convolve each image by the fitted clean beam before computing *ρ*_NX_. Throughout this work, we use *ρ*_NX_ to evaluate the inter-method consistency in resulting image reconstructions, often between the GenDIReCT and EHTC fiducial imaging pipelines or simulated images. As there is no ground-truth reconstruction for M87*, the *ρ*_NX_ metric does not constitute an independent ground-truth validation metric but as an image-domain similarity metric between various imaging algorithms.

### Image-domain feature extraction

The image-domain feature extraction method presented here follows the framework established by the EHTC in their initial analysis of M87* (ref. ^3^). We outline the reproduction of this algorithm below. Given any plausibly ring-like image, we first search for the ring centre, (*x*_0_, *y*_0_), by evaluating all candidate centroid positions for the one that minimizes the normalized radial peak dispersion

4 $$({x}_{0},{y}_{0})={\rm{argmin}}\,{\left[\frac{{\sigma }_{r}(x,y)}{{\bar{r}}_{{\rm{pk}}}(x,y)}\right]}_{(x,y)},$$

in which ${\bar{r}}_{{\rm{pk}}}(x,y)$ is the radius of peak brightness averaged over all angles, as

5 $${\bar{r}}_{{\rm{pk}}}(x,y)={\langle {{\rm{argmax}}}_{r}[I(r,\theta ;x,y)]\rangle }_{\theta \in [0,2{\rm{\pi }}]},$$

in which *I*(*r*, *θ*; *x*, *y*) is the intensity distribution of the image in radial coordinates, centred on (*x*, *y*). The dispersion, *σ*_*r*_(*x*, *y*), is the corresponding standard deviation of *r*_pk_(*θ*; *x*, *y*) across all angles, *θ*. The ring diameter and its uncertainty can then be identified from ${\bar{r}}_{{\rm{pk}}}(x,y)$ and *σ*_*r*_(*x*, *y*), respectively, given the measured ring centre, (*x*_0_, *y*_0_).

Once the ring centre is determined, most other characteristic features can be extracted directly from the polar image, including: the ring width *w*_r_, orientation *η*, azimuthal asymmetry *A* and central brightness fraction *f*_c_. The ring width, *w*_r_, is determined by measuring the mean FWHM at each radial slice for the given ring centre,

6 $${w}_{{\rm{r}}}={\langle {\rm{FWHM}}[I(r,\theta )-{I}_{{\rm{floor}}}]\rangle }_{\theta },$$

in which the floor value *I*_floor_ = ⟨*I*(*r* = 2*r*_pk_, *θ*)⟩_*θ*_ is used to correct for any non-zero residual flux outside the ring. The uncertainty on the ring width, *σ*_*w*_, is measured by the standard deviation in FWHM across all *θ*.

The orientation angle, *η*, of the ring (measured east of north) is the circular mean of the first angular mode from *r*_in_ = *r*_pk_ − *w*_r_/2 to *r*_out_ = *r*_pk_ + *w*_r_/2, as

7 $$\eta ={\langle \arg \left[{\int }_{0}^{2{\rm{\pi }}}I(\theta ){{\rm{e}}}^{{\rm{i}}\theta }{\rm{d}}\theta \right]\rangle }_{r\in [{r}_{{\rm{in}}},{r}_{{\rm{out}}}]},$$

and the corresponding uncertainty, *θ*_*η*_, is the standard deviation of the measured angles for *r* ∈ [*r*_in_, *r*_out_].

The azimuthal asymmetry, *A*, is the mean of the normalized amplitude in the first angular mode from *r*_in_ to *r*_out_, which is

8 $$A={\langle \frac{|{\int }_{0}^{2{\rm{\pi }}}I(\theta ){{\rm{e}}}^{{\rm{i}}\theta }{\rm{d}}\theta |}{{\int }_{0}^{2{\rm{\pi }}}I(\theta ){{\rm{e}}}^{{\rm{i}}\theta }{\rm{d}}\theta }\rangle }_{r\in [{r}_{{\rm{in}}},{r}_{{\rm{out}}}]},$$

in which *σ*_*A*_ is the standard deviation in the measured asymmetry over *r* ∈ [*r*_in_, *r*_out_]. Perfect azimuthal symmetry corresponds to a value of *A* = 0, whereas a delta function with all flux concentrated at a single orientation angle corresponds to a value of *A* = 1.

**Extended Data Fig. 5:**
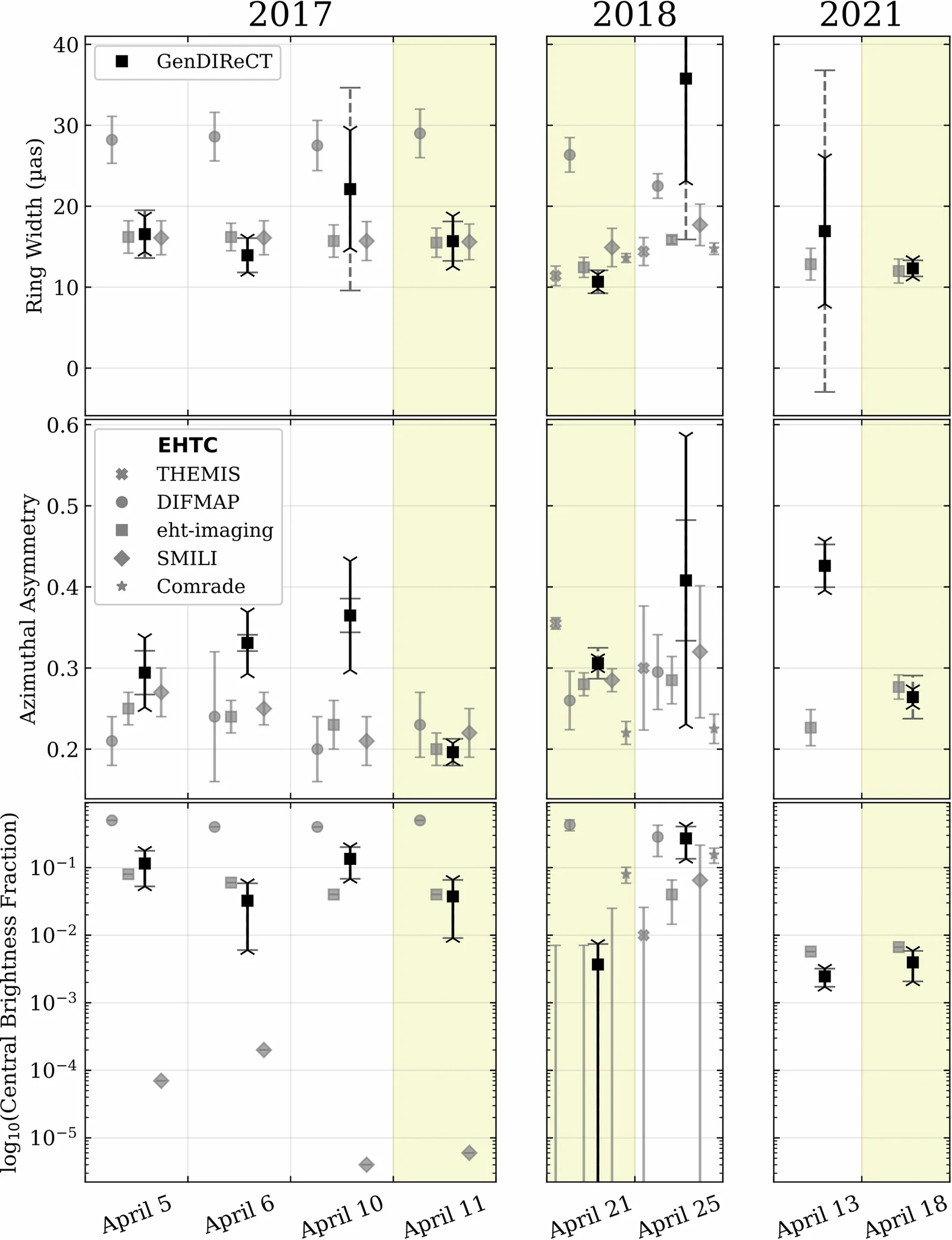
Measured ring width, azimuthal asymmetry and central brightness fraction. Similar to Fig. 2, illustrating a comparison of the ring properties measured from image-domain feature extraction applied to our reconstructions with values from the EHTC, separated by observation epoch.

Finally, the central brightness fraction, *f*_c_, is the ratio between the mean interior depression brightness to the mean ring surface brightness, as

9 $${f}_{{\rm{c}}}=\frac{{\langle I(r,\theta )\rangle }_{\theta ,r\in [0,0.2{r}_{{\rm{pk}}}]}}{{\langle I({r}_{{\rm{pk}}},\theta )\rangle }_{\theta }},$$

in which the interior brightness is measured from the average flux in a small disk around the ring centre. Although we do not define an uncertainty for a single measurement of *f*_c_, we derive its uncertainty from several measurements across the three different fields of view used in GenDIReCT reconstruction. Figure 2 presents the measured ring diameter and orientation across all datasets considered in this work. We present the measurements for ring width, azimuthal asymmetry and central brightness fraction in Extended Data Fig. 5.

The EHTC derived ring parameters using the REX module implemented as part of eht-imaging. Ring properties from three different imaging algorithms (DIFMAP^37^, eht-imaging^28^ and SMILI^66^) were reported for the 2017 data^3^, two Bayesian imaging algorithms (THEMIS^86^ and Comrade^87,88^) were included for the 2018 datasets^33^ and for 2021 data, one further CLEAN-based algorithm (GPCAL^89^) and two further regularized maximum likelihood algorithms (DOG-HIT^67^ and MOEA/D (ref. ^35^)) were added. However, for the 2021 datasets, the EHTC used an alternative template-matching image-domain feature extraction algorithm^34^. As such, to maintain methodological consistency, we have reproduced a single eht-imaging reconstruction to use as the EHTC reference.

When comparing ring diameter between datasets, image reconstruction algorithms and across multiyear epochs, there is potential for systematic bias arising from the finite-resolution effect, in which variations in ring width can shift the apparent diameter derived from a direct image-domain feature analysis. Recent EHTC analyses use a forward-modelling procedure to mitigate diameter–width degeneracy^34^. In this work, we choose to maintain methodological consistency across all reconstructions by deriving diameters directly from the image domain without further adjustment. Therefore, the apparent variation observed between imaging algorithms and individual datasets may be influenced by the finite resolution of each imaging method. Despite this, the apparent ring diameter derived from our reconstructions remains self-consistent across all independent datasets.

### Geometric modelling

Our complementary Bayesian analysis uses the geometric xsringauss model^4,90^, which was designed to simulate the appearance of quasi-Kerr black hole accretion disk radio images^91^. The advantage of geometric modelling is that visibilities can be derived analytically from simple algebraic expressions and fit to the observed measurements, which substantially reduces the computational requirement of modelling compared with complex and highly nonlinear physical models, particularly when applied with Bayesian sampling algorithms. Moreover, as long as the object is plausibly described by the model, Bayesian sampling methods applied with geometric models enable direct inference of key features of interest from the observed object with associated uncertainties. In this section, we provide a brief description of the xsringauss model, the extra components we included to form the model for fitting and the complete analytical visibility function. We develop the BASIS^92^ software package for Bayesian analysis with the xsringauss model independent from existing software from the EHTC. For the following discussion, we use ${\mathcal{F}}$ to denote the forward Fourier transform operator.

The xsringauss model is parameterized with the following variables^4^. The outer disk radius, *R*_out_, and inner disk radius, *R*_in_, create the centralized ‘top-hat crescent’, in which the offset of the inner disk centroid is controlled by the eccentricity, *ϵ* ∈ [0, 1]. At *ϵ* = 0, the centres of both disks are co-located at the origin and at the maximum eccentricity, *ϵ* = 1, the edge of the inner disk meets the edge of the outer disk. The fading parameter, *f* ∈ [0, 1], controls the surface brightness gradient across the crescent, with the maximum brightness located at the tip of the crescent, at which the crescent width is maximum. When *f* = 0, the crescent surface brightness is uniform and when *f* = 1, the surface brightness fades to zero at *x* = −*R*_out_. Note that we have deliberately adjusted the definition of fading from its original conception^90^.

The xsringauss model also includes an elliptical Gaussian centred on the inner disk edge, at which the crescent is widest. Its flux is determined by a fraction, *f*_g_, of the total flux and its width is set by *σ*_e_, with axial ratio, *a*_g_. The longest axis is always oriented perpendicular to the crescent’s axis of symmetry. A circular Gaussian with flux *V*_f_ and width *σ*_f_ centred at *x*_0_ represents the floor component internal to the crescent. To complete the base xsringauss model, we blur the image with a circular Gaussian kernel of width *σ*_b_ and rotate the entire image by angle *ϕ*.

Further components include a larger-scale circular background Gaussian of flux *V*_bg_ and width *σ*_bg_ centred at the origin, which is added to include short-spacing flux that is not examined by most of the long-baseline array. Finally, two more nuisance elliptical Gaussians are included, each with independent parameters for flux, width, axial ratio, position angle, angular offsets and Gaussian blur, to capture extraneous amorphous emission that is not modelled by the ring alone and as further degrees of freedom to absorb the effects from measurement errors and systematic uncertainties.

In the Fourier domain, the composite model is a linear combination of elementary components with analytic Fourier transforms. The general Fourier transform (represented by tilde) of a disk with radius *R* and amplitude *V* is given by

10 $$\tilde{D}(\rho )=\frac{{VR}}{\rho }{J}_{1}(2{\rm{\pi }}\rho R),$$

in which *ρ* is the Fourier-domain radial coordinate and *J*_1_ is a first-order Bessel function of the first kind. The Fourier transform of a Gaussian with flux *V* and width *σ* is,

11 $$\tilde{G}(\rho )=V{{\rm{e}}}^{-2{{\rm{\pi }}}^{2}{\sigma }^{2}{\rho }^{2}},$$

in which the Fourier transform of asymmetric Gaussians are determined by exploiting the similarity property of the Fourier transform, that is, ${\mathcal{F}}(G({ax},{by}))=\frac{1}{|{ab}|}\tilde{G}\left(\frac{u}{a},\frac{v}{b}\right)$. Multiplication in the image domain of the crescent by the sloped plane is represented in the Fourier domain by taking a derivative

12 $${\mathcal{F}}(xF(x))=\frac{i}{2{\rm{\pi }}}\frac{{\rm{d}}}{{\rm{d}}s}\tilde{F}(s),$$

in which, for our specific linear slash function, *l*(*x*), the corresponding operation in the Fourier domain is

13 $${\mathcal{F}}(l(x)F(x))=h\,\left(1-\frac{f}{2}+\frac{i}{2{\rm{\pi }}{R}_{{\rm{out}}}}\frac{f}{2}\frac{{\rm{d}}}{{\rm{d}}s}\right)\tilde{F}(s),$$

in which the derivatives of Bessel functions of the first kind, *J*_*n*_, can be expressed in terms of other Bessel functions, *J*_*n*±1_, as

14 $$\frac{{\rm{d}}}{{\rm{d}}\rho }{J}_{1}(2{\rm{\pi }}\rho R)={\rm{\pi }}R[\,{J}_{0}(2{\rm{\pi }}\rho R)-{J}_{2}(2{\rm{\pi }}\rho R)],$$

and *h* is responsible for normalizing the source to the desired total flux, *V*, which is dependent on the source structure. For the xsringauss model, the normalization factor is

15 $$h=\frac{2V}{{\rm{\pi }}\,\left[{R}_{{\rm{out}}}^{2}(2-f)+{R}_{{\rm{in}}}^{2}\left(f\left(1+\frac{{x}_{0}}{{R}_{{\rm{out}}}}\right)-2\right)\right]}.$$

**Extended Data Fig. 6:**
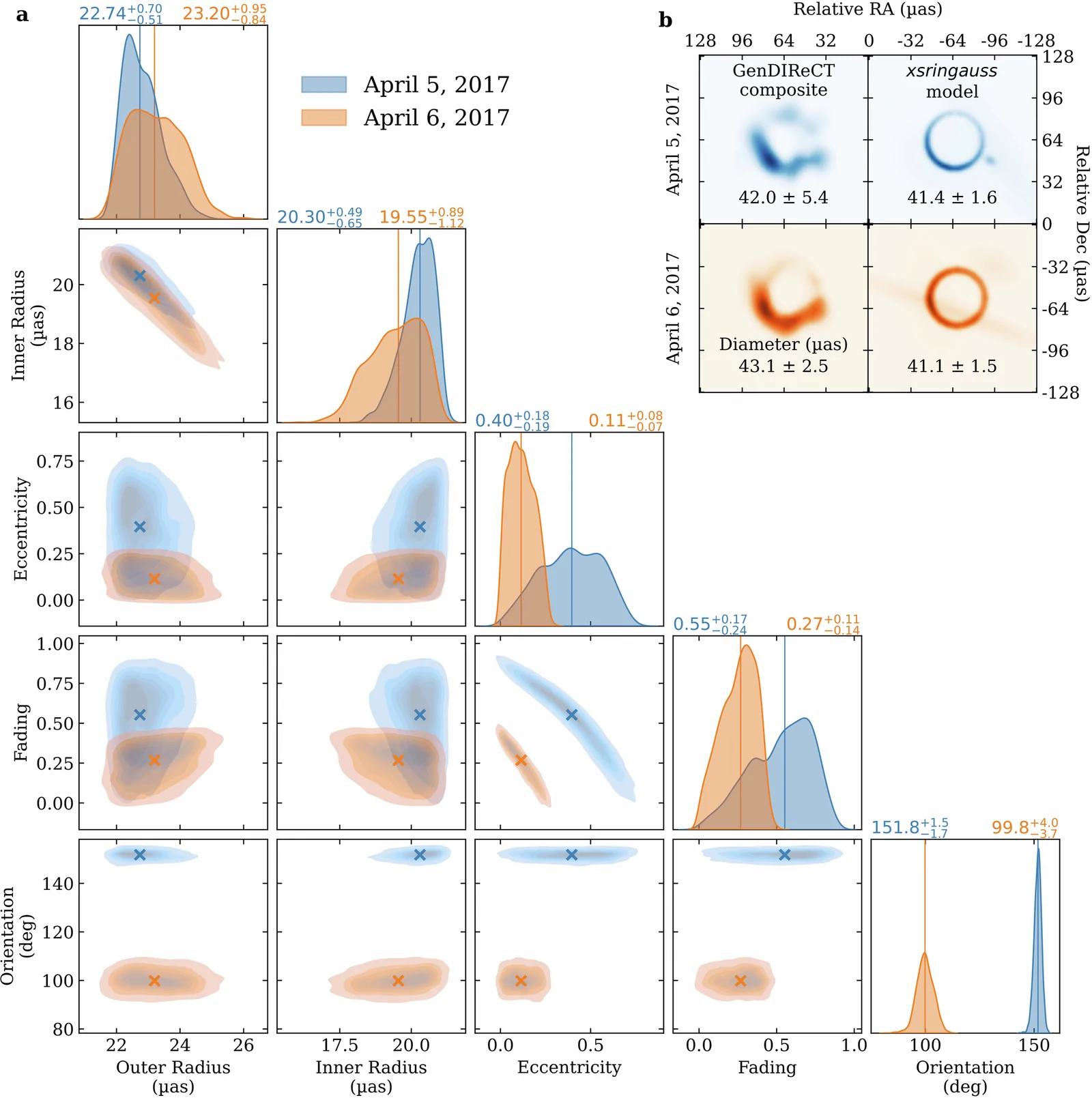
Posterior covariance from xsringauss geometric modelling on the 5 April 2017 and 6 April 2017 datasets. One-dimensional and two-dimensional marginalised posterior distributions with representative composite images and median sky models similar to Fig. 3.

**Extended Data Fig. 7:**
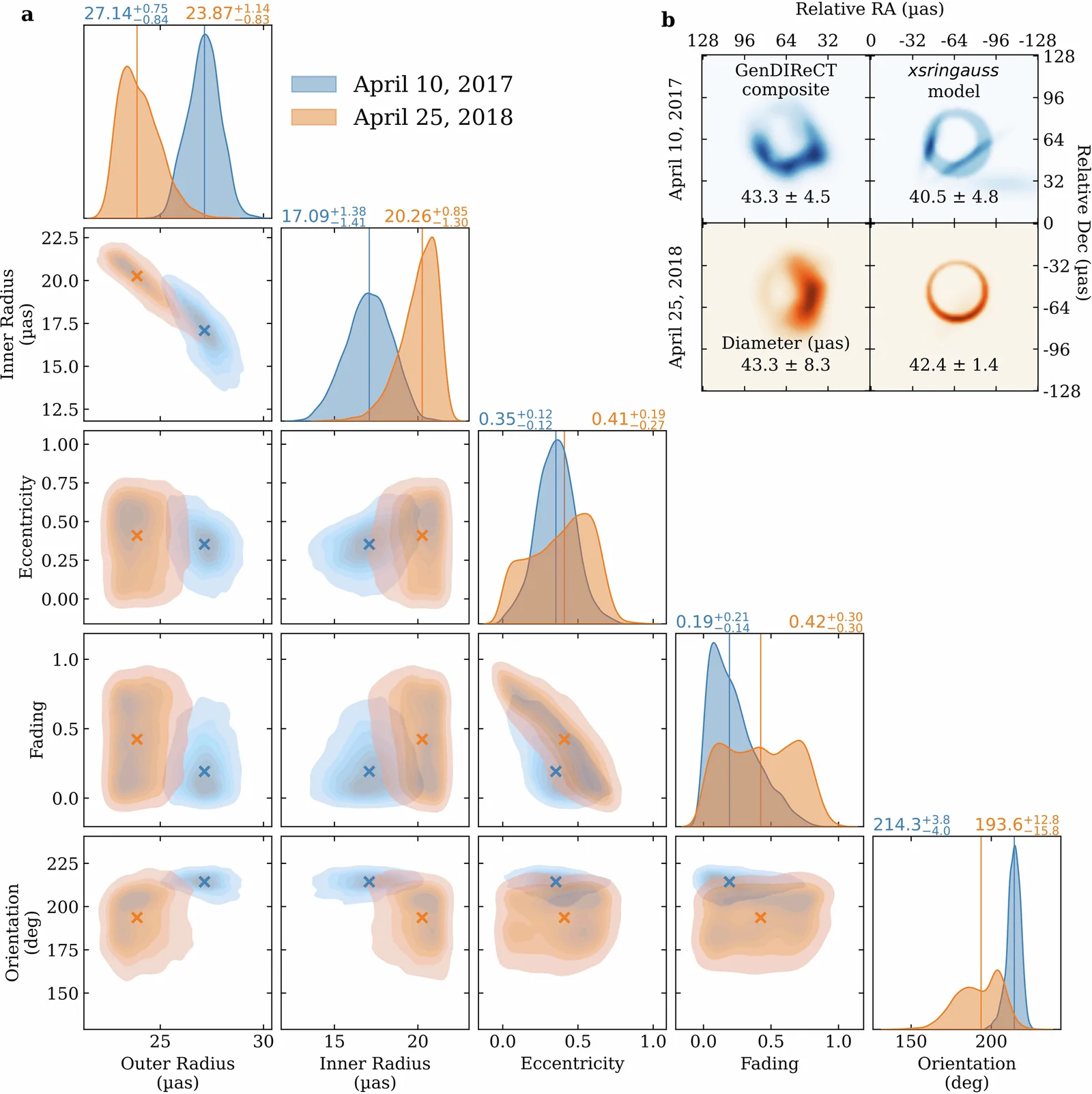
Posterior covariance from xsringauss geometric modelling on the 10 April 2017 and 25 April 2018 datasets. One-dimensional and two-dimensional marginalised posterior distributions with representative composite images and median sky models similar to Fig. 3.

**Extended Data Fig. 8:**
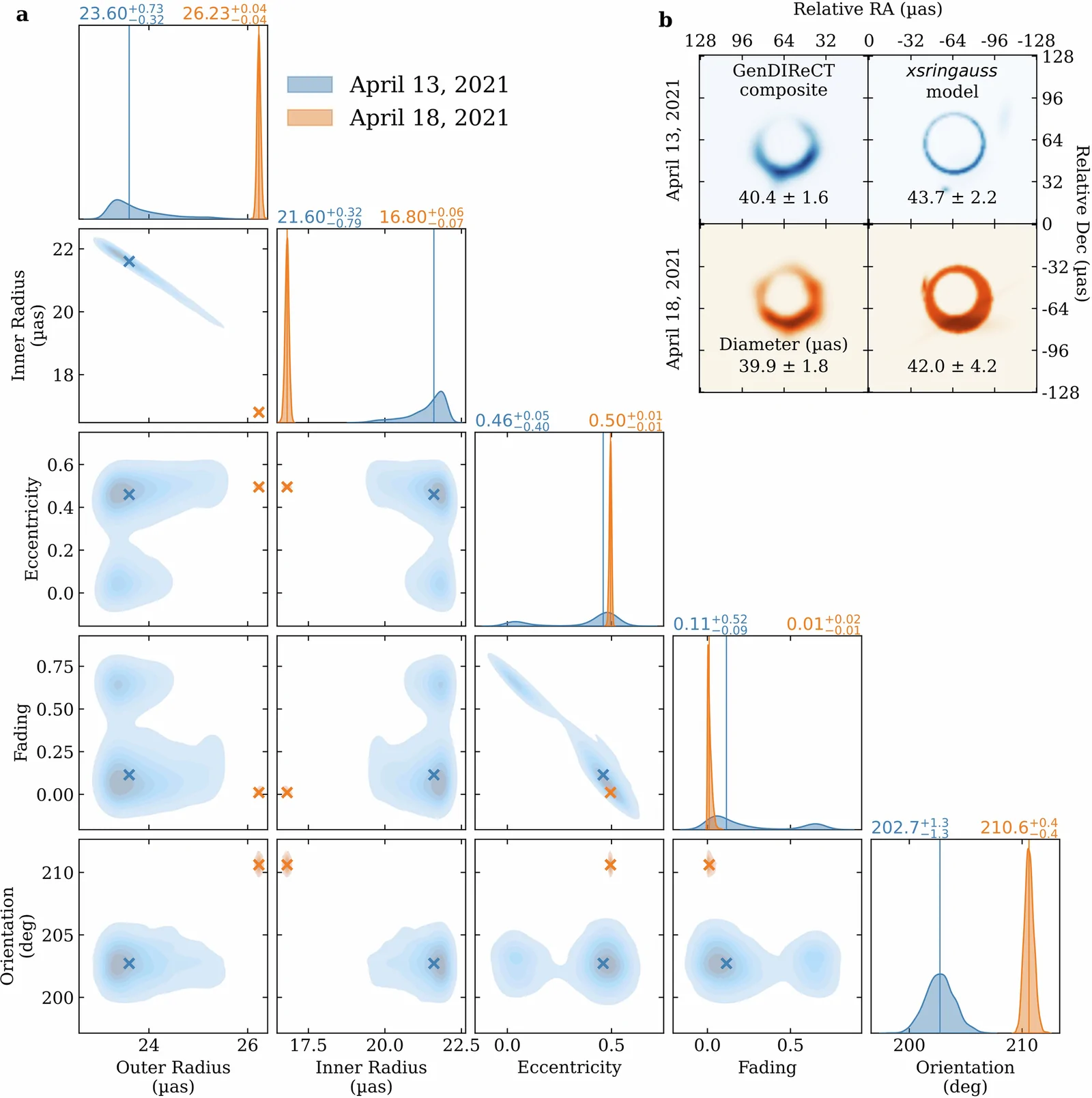
Posterior covariance from xsringauss geometric modelling on the 13 April 2021 and 18 April 2021 datasets. One-dimensional and two-dimensional marginalised posterior distributions with representative composite images and median sky models similar to Fig. 3.

Each image component can be further independently augmented by translation, rotation and Gaussian blur. In general, image translations are represented with an extra phase term in the Fourier domain, ${{\rm{e}}}^{{\rm{i}}2{\rm{\pi }}({l}_{0}u+{m}_{0}v)}$, in which (*l*_0_, *m*_0_) denotes the translation in (*l*, *m*) coordinates. Image rotation is modelled by a rotation of the *uv* coordinates. Then, by the convolution theorem, blurring in the image domain with a circular Gaussian kernel is a pointwise multiplication in the Fourier domain by ${{\rm{e}}}^{-{\rm{i}}2{{\rm{\pi }}}^{2}{\sigma }_{{\rm{b}}}^{2}{\rho }^{2}}$, in which *σ*_b_ is the width of the blurring kernel. The final image model and its corresponding analytic Fourier transform is described by 28 parameters. Throughout this work, the xsringauss model is fit to observed data with DYNESTY, a Bayesian dynamic nested sampling algorithm, adopting uniform priors for all parameters. The final fitted image model is formed from the median value of the one-dimensional marginalized posteriors for each parameter. The xsringauss model parameter covariance plot for the highest-quality datasets of 2017 and 2018 is shown in Fig. 3 and we present covariance plots for all other datasets in Extended Data Figs. 6–8. We then apply the image-domain feature extraction analysis on samples from the posterior to measure all characteristic ring parameters (Extended Data Table 1).

### Inferring the black hole mass

Inferring the gravitational radius of the black hole from the measured ring diameter requires independently calibrating each imaging method with synthetic data from simulated GRMHD models of lensed black hole accretion emission^4^. One common simplification is that the observed size of the reconstructed ring should follow a simple scaling relationship with the gravitational radius, *d*_ring_ = *αθ*_g_. By calibrating to a large ensemble of simulations with different black hole spins, the EHTC determined the calibration factor, *α*, from the image-domain feature extraction method to be between 10.67 and 11.01, in which the listed range is the result of variations from different image reconstruction algorithms. For geometric modelling methods, the *α* calibration factor ranges between 11.01 and 12.06. The median of all *α* calibrations is *α* ≃ 11.0, which is the calibration factor used by the EHTC for methods that have not been independently calibrated^33^. Therefore, we also provisionally apply *α* ≈ 11.0 as our calibration factor in this study.

The associated theoretical uncertainty in the calibrated scaling factor, which arises from recognizing the dataset as a single sample from a dynamic system with unknown physical properties, is *σ*_*α*_ = 0.4 (ref. ^4^). In this work, we also use the mean M87* distance measure from the EHTC, which is $16.{8}_{-0.7}^{+0.8}\,{\rm{Mpc}}$ (ref. ^3^) based on studies of high-resolution resolved stellar distribution fluctuations^13,93^ and the tip of the red giant branch standard candle method^14^.

The prior expectation for the mass of the central black hole in M87 is primarily sourced from high-quality gas^12^ and stellar^11^ dynamical estimates, which measured ${M}_{{\rm{gas}}}=3.{5}_{-0.7}^{+0.9}\times 1{0}^{9}{M}_{\odot }$ and *M*_star_ = (6.6 ± 0.4) × 10^9^ *M*_⊙_, respectively. The corresponding gravitational radii are ${\theta }_{{\rm{gas}}}=2.{1}_{-0.4}^{+0.5}\,{\rm{\mu }}{\rm{as}}$ and *θ*_star_ = 3.9 ± 0.3 μas if we use a distance measure of $16.{8}_{-0.7}^{+0.8}\,{\rm{Mpc}}$. Using the median *α* = 11.0 ± 0.4 calibration factor and theoretical uncertainty, the prior ring diameters are ${d}_{{\rm{gas}}}=22.{6}_{-4.7}^{+5.9}\,{\rm{\mu }}{\rm{as}}$ and ${d}_{{\rm{star}}}=42.{7}_{-3.6}^{+3.5}\,{\rm{\mu }}{\rm{as}}$. We therefore estimate a black hole mass of ${M}_{{\rm{BH}}}=6.{5}_{-0.4}^{+0.5}\,(11.0/\alpha )\times 1{0}^{9}\,{M}_{\odot }$ with GenDIReCT’s independently measured mean ring diameter. The full range of *α* = 10.67–12.06 calibration factors implies an estimated black hole mass in the range *M*_BH_ = (6.0–6.7) × 10^9^ *M*_⊙_. On this basis, we conservatively estimate our black hole mass as *M*_BH_ = (6.5 ± 0.5) × 10^9^ *M*_⊙_.

### Synthetic M87* images

To compare the reconstructed composites to synthetic M87* images, we retrieve publicly available GRMHD data (https://thz.astro.illinois.edu/resources.html) from the University of Illinois survey^41^, which includes fluid data for 1,000 time steps (in *R*_g_/*c*) in the quasi-stationary equilibrium state and five black hole spin prescriptions (*a*⁎ = −0.94, −0.5, 0, +0.5, +0.94), evolving under the magnetically arrested disk or standard and normal evolution regimes. In this work, we consider only the magnetically arrested disk fluid models, which is the more likely evolutionary scenario for M87* based on jet power constraints^94^.

Synthetic 230-GHz images of M87* are produced using the general relativistic radiative transfer IPOLE algorithm^40^ for all time steps, spins and inclination angles between 5° and 90° with a step size of 1°. Note that we use a coupled electron temperature model characterized by *R*_high_ = 40, which determines the relationship between the ion and electron temperature. Although *R*_high_ is a free parameter, the time-averaged magnetically arrested disk images are nearly independent of *R*_high_ and are sensitive primarily on black hole spin for a fixed inclination^94^. In this work, we compare all beam-convolved synthetic images with the reconstructed M87* composites in the image domain using the normalized cross-correlation metric after correcting for the observer position angle and making linear adjustments to spatial scale, for which both the rotation and scale adjustments of the synthetic images are chosen independently for each parameter combination of spin, inclination and time step, to maximize the resulting normalized cross-correlation comparison with the composite reconstructions. Simulations in ideal GRMHD models are scale-agnostic, enabling such adjustments to be made without affecting the underlying physics^41,95^. We also repeat our analysis for *R*_high_ = 80, presenting the figure in the GenDIReCT repository, and find a mild roughly 2° shift towards lower inclinations.

## Online content

Any methods, additional references, Nature Portfolio reporting summaries, source data, extended data, supplementary information, acknowledgements, peer review information; details of author contributions and competing interests; and statements of data and code availability are available at https://doi.org/10.1038/s41586-026-11241-9.

## Supplementary information

Peer Review File

## Figures and Tables

**Extended Data Table 1:**
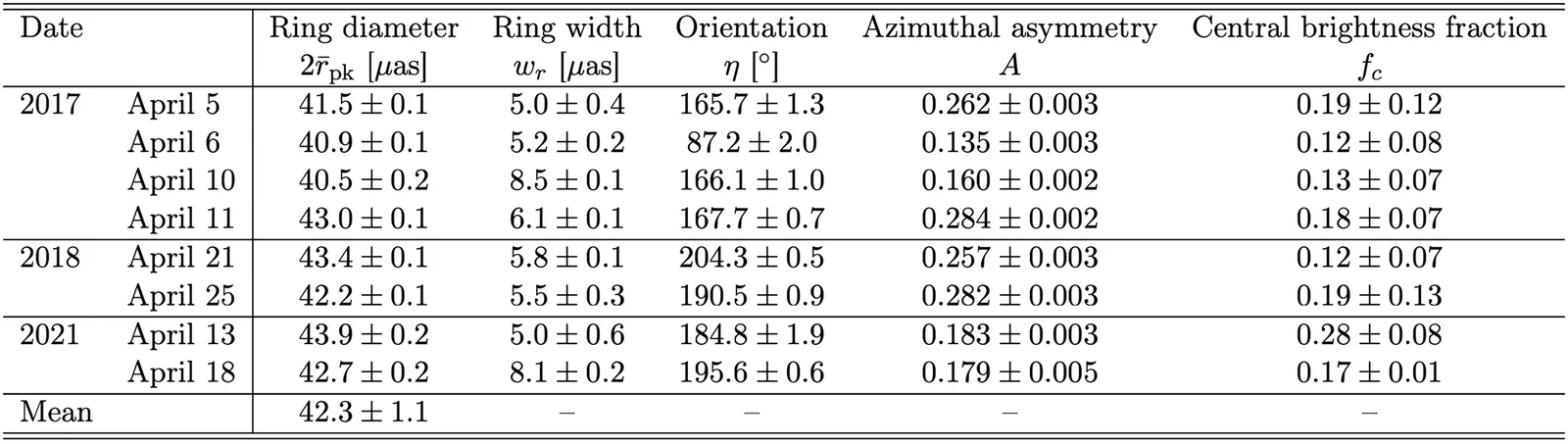
Similar to Table 1 but with ring properties measured from geometric modelling of the closure invariants datasets from each independent epoch

For all parameters, we present the mean and standard deviation derived from sampling the posterior and applying image-domain feature extraction.

## Data Availability

The science-ready observational data underlying this paper are publicly available from the Event Horizon Telescope Collaboration website (https://eventhorizontelescope.org/), namely, EHT Data Product Codes 2019-D01-01, 2024-D01-01 and 2026-D01-01. Post-processed data products or image reconstructions can be located in the GenDIReCT code repository^96^. Software packages used in this study include NumPy^97^, SciPy^98^, PyTorch^99^, eht-imaging^28^, GenDIReCT^96^, BASIS^92^, ClosureInvariants^100^, DYNESTY^38^, BILBY^101^, IPOLE^40^ and Matplotlib^102^.

## Code availability

Code for GenDIReCT^96^, geometric modelling with image-domain feature extraction^92^ and the ClosureInvariants formalism^97^ are publicly available.
