# Supplementary material for: Independent interferometric observable confirms black hole shadow in M87*
Source: Nature. 2026 Nov 11;659(8140):317–23. doi: 10.1038/s41586-026-11241-9 (PMC13754589; doi:10.1038/s41586-026-11241-9)
Supplement: Supplementary file 1 [file 41586_2026_11241_MOESM1_ESM.pdf]

nature portfolio

Peer Review File

New interferometric observable confirms black hole shadow in M87

Corresponding Author: Dr Samuel Lai

Any redactions in this file are there to maintain patient confidentiality, the confidentiality of unpublished data, or to remove third-party material.

This file contains all reviewer reports in order by version, followed by all author rebuttals in order by version.

Version 0:

Reviewer comments:

Referee #1

(Remarks to the Author)
I have reviewed this manuscript, which presents an application of the GenDIReCT imaging algorithm to EHT 2017 and 2018 M87* data, using interferometric closure invariants as the sole data term. The work addresses an important problem and the core methodology is sound. I consider the central claim broadly supportable, but several specific issues require clarification or revision before the paper is suitable for publication in Nature. My detailed comments follow.

A. Summary of the Key Results

The authors apply GenDIReCT, a novel imaging algorithm utilising a conditional latent diffusion model and multi-layer convolutional neural network trained on closure invariants and non-astronomical images, to six epochs of EHT observations of M87* (four nights from April 2017 and two nights from April 2018). The method reconstructs images without requiring explicit gain calibration, instead operating directly on interferometric closure invariants — quantities that are algebraically immune to station-based multiplicative gain errors. The reconstructed images consistently recover a ring-like morphology. Averaging ring diameter measurements across all six epochs yields an angular diameter of 42.9 ± 0.4 μas, from which the authors derive a black hole mass consistent with the EHTC's 2019 estimate. Data-fidelity and image-similarity metrics are reported, with the best-performing epoch (April 11, 2017) achieving χ_CI^2= 1.1 and ρ_NX image-domain similarity scores of 0.98, 0.94, and 0.93 at field-of-view scales of 128×128, 225×225, and 450×450 μas respectively, evaluated against the EHTC's fiducial reconstruction.

B. Originality and Significance

The application of a generative, closure-invariant-based imaging approach to real EHT M87* data represents a meaningful contribution to the field of VLBI imaging. Demonstrating that a method operating without any gain calibration can recover morphological features consistent with the EHTC's calibrated reconstructions is a valuable cross-check, and the methodology is timely given ongoing development of future EHT observations. The GenDIReCT method itself was published in Lai et al. (2025, PASA); the novelty of the present manuscript lies in its application to real M87* data and in the independent confirmation it provides.

The reference list includes Jennison (1958) and Twiss et al. (1960) among refs 20–26, which is appropriate. However, these are cited collectively in a single bracket alongside more recent developments without distinguishing their individual historical contributions. Separately, while the authors do not claim novelty for the closure concept itself, the title's phrasing — "a new interferometric observable" — may be read by a general Nature readership as implying that the observable itself is new, rather than its application within a generative imaging framework. A brief clarification in the introduction distinguishing the long history of closure quantities from the specific novelty of this work would be beneficial.

The claim of independent confirmation is well-framed: the authors present their result as independent both from the EHTC's calibrated analysis and from alternative morphological interpretations of M87* (e.g. Miyoshi et al. 2022, 2024), by virtue of operating on calibration-immune observables without reference to either group's methodology. This is a meaningful and

clearly defined form of independence that the paper defends credibly.

C. Data and Methodology

The core methodology is well-motivated and the data (EHT 2017 and 2018 M87* observations) are appropriate and well-established. I have several specific concerns about the methodological presentation.

Description of the generative model and architecture. The paper would benefit from a more self-contained description of the conditional latent diffusion model architecture and its training procedure. In particular, the rationale for key architectural choices — such as CNN details, number of attention layers, latent space dimensionality — is not provided. Similarly, the paper states that augmented CIFAR-10 images and simple geometric shapes are included in the training data to improve generalisation to non-astrophysical morphologies, but no example images from this augmented dataset are shown. Including representative training samples would strengthen the reader's understanding of the ring-morphology agnosticism encoded in the model.

Median image construction and uncertainty quantification. Several aspects of the ensemble aggregation procedure require clarification. First, the total number of diffusion samples drawn per reconstruction is not stated; the authors should specify this explicitly. Second, the rationale for adopting the pixelwise median rather than the mean as the summary reconstruction is not discussed. Other diffusion-based imaging methods in the literature use the posterior mean; the authors should justify this choice, particularly since the median and mean coincide only for symmetric unimodal distributions and may diverge under multimodal posteriors. Third, and relatedly, no pixel-level uncertainty map is presented. The pixelwise standard deviation of the ensemble — a natural and commonly used uncertainty estimate for generative ensembles — is absent. The authors should explain why the standard deviation was not used, and either provide it or justify its omission.

D. Appropriate Use of Statistics and Treatment of Uncertainties

χ_CI^2 as a fidelity metric. The reduced chi-squared statistic χ_CI^2 measures consistency with the observed closure invariants. A value near unity confirms that the reconstruction is self-consistent in the closure domain but does not verify that the recovered morphology is correct in an absolute sense. The authors should be explicit that χ_CI^2 ≈ 1 is a necessary but not sufficient condition for a faithful reconstruction.

ρ_NX as a similarity metric. The ρ_NX scores reported are cross-correlations between GenDIReCT reconstructions and the EHTC's fiducial images. These measure inter-method consistency, not ground-truth fidelity. The authors should state this distinction clearly: a high ρ_NX indicates that GenDIReCT recovers similar features to RML methods, which is informative, but it does not constitute independent ground-truth validation.

E. Conclusions: Robustness, Validity, Reliability

The paper's central conclusion — that an independent analysis of EHT M87* data confirms a ring-like black hole shadow morphology with an angular diameter of 42.9 ± 0.4 μas — is well-supported by the presented reconstructions and metrics. The consistency of the ring morphology across all six epochs, and the stability of ρ_NX scores across field-of-view scales, provides genuine support for the robustness of the morphological recovery. The method's agreement with the EHTC's fiducial reconstruction, achieved without any gain calibration, is a credible and meaningful result.

The derived black hole mass rests on the provisional adoption of α ~ 11.0 from the EHTC, which the authors themselves acknowledge has not been independently calibrated for GenDIReCT. The mass measurement should therefore be presented explicitly as provisional, with the associated systematic uncertainty acknowledged. The morphological confirmation — the primary scientific contribution of this work — stands independently of this limitation and is the stronger of the two claims.

F. Suggested Improvements

I request the following revisions:

1. Architecture description and training data examples. Please provide a more complete description of the diffusion model architecture, including the rationale for key design choices. Include representative examples from the augmented CIFAR-10 and geometric shape training datasets to illustrate the morphological diversity of the training distribution.

2. Ensemble aggregation: sample count, choice of median, and uncertainty maps. Please state explicitly the total number of diffusion samples drawn per reconstruction. Provide a justification for the use of the pixelwise median rather than the mean, with reference to the properties of the posterior distribution. Please also provide pixelwise standard deviation maps as a natural uncertainty quantification, or justify their omission and describe what uncertainty estimate is being offered in their place.

3. Provisional mass estimate. The mass estimate should be clearly presented as provisional, with an explicit acknowledgement that the adopted α ~ 11.0 has not been independently calibrated for GenDIReCT. Where feasible, the authors should propagate the range α = 10.67–12.06 as a systematic uncertainty on the quoted mass.

Comments on code availability are provided separately in the dedicated code review field.

G. References: Appropriate Credit to Previous Work

The reference list is broadly appropriate. I note two specific concerns. First, refs 20 (Jennison 1958) and 21 (Twiss et al. 1960) are cited collectively within the bracket refs 20–26 without distinguishing their historical roles as the originators of closure phases and closure amplitudes respectively. Given the paper's reference to a "new" observable in the title, explicit historical attribution in the text would strengthen clarity. Second, refs 27 and 28 (Wilkinson et al. 1977; Pearson et al. 1981) are cited to support the claim that closure invariants "underpin high-fidelity morphology reconstructions." These are early VLBI imaging works that do not specifically demonstrate closure-invariant-based reconstruction. More directly relevant citations for this specific claim would be Chael et al. (2018, ref 36) or Thyagarajan et al. (2024, ref 31), which explicitly address imaging from closure quantities.

H. Clarity and Context

The abstract and title present the independent confirmation claim clearly, and the paper's overall structure is logical. The precise sense of "independence" — from gain calibration, from the EHTC's methodology, and from alternative morphological interpretations — should be stated explicitly in the abstract and conclusions rather than left implicit.

The paper's description of GenDIReCT as operating "without a prior" warrants revision. The denoising diffusion framework requires sampling from an isotropic Gaussian at the terminal diffusion timestep, which constitutes an explicit prior, and the learned score function encodes an implicit morphological prior from the training distribution. The correct and defensible claim is that GenDIReCT does not require a user-specified, hand-tuned image initialisation at inference time — a genuine and useful property that should be stated accurately.

I am willing to reconsider this manuscript following revisions addressing the concerns detailed above.

(Remarks on code availability)
The repository (https://github.com/samlaihei/GenDIReCT) currently presents several barriers to reproduction:
(i) model weights are tracked via Git LFS but this requirement is not documented in the README, causing silent pointer-file checkout and a cryptic UnpicklingError at runtime;
(ii) the ClosureInvariants library installation is not described;
(iii) the image-domain feature extraction code referenced in the paper does not appear to be present in the repository;
(iv) the python scripts are generally not well documented; and
(v) no instructions are provided for reproducing the paper's primary results.

The README should be revised to address the first three points, and the missing code should be included or its absence clearly explained. The python scripts should be documented more comprehensively, and an instruction in README or a Jupyter notebook tutorial should be provided for reproducing the paper's primary results.

Referee #2

(Remarks to the Author)
I co-reviewed this manuscript with one of the reviewers who provided the listed reports.

(Remarks on code availability)

Referee #3

(Remarks to the Author)
The paper "Independent confirmation of the M87* black hole shadow with a new interferometric observable" presents a new, independent reconstruction of EHT 2017 and 2018 observations of M87* with a novel analysis algorithm GeNDIRECT, which has already been validated and applied to real data in several other papers. In reproducing these important images with a completely independent analysis method, this paper is an important addition to the literature. The approach is well-documented and sound; the underlying method having been validated in several previous papers; the software and underlying data are publicly available. The paper explores uncertainties in the image reconstructions well, particularly in the Methods section. The paper is overall clear and well presented. However, I have several comments on the presentation and claims throughout that I would like the authors to address.

Pg 1: In paragraph 1, it would help to introduce M87*'s mass and distance in order to quantify the size estimate for the BH shadow image and justify the need to go to mm wavelength VLBI.

Pg 1: Since the paper rightly notes that instrument calibration (phase and amplitude) is as significant as sparse u,v coverage in setting the difficulty of the M87* imaging problem, it would be useful to describe these challenges for mm VLBI in one additional sentence in paragraph 2.

Pg 1: In describing the "divergent representations of M87*'s fine-scale features," it would be useful to have a little bit more discussion on what differences between representations are acceptable consequences of solving the ill-posed imaging problem and what are concerning.

Pg 1: “Iconic ring structure” → I would choose a different adjective here

Pg 2: I think the paper would benefit from revisiting the summary of closure invariants presented here. The paper says that closure invariants unify the traditional closure phases and amplitudes, but it is not clear from the summary if they carry more information than these data products, which were used in the first EHT papers.

Pg 2: “Unlike other machine-learning based radio interferometric imaging algorithms….” → is the key differences in these other algorithms cited that they use different data products (visibilities or traditional closure quantities) rather than the paper’s closure invariants? Or is it the type of prior?

Pg 2: “we aggregate a large population of reconstructions” → It would be useful to indicate in the main text what parameters in the algorithm determine these different reconstructions.

Fig 1: the main lobe of the PSF/beam is indicated in these panels, but the image is clearly not convolved with it. That is justifiable , but it would be useful to explain that choice. For comparison to the main EHT result images, it would be useful to also show a version convolved with the same fiducial beam as in those papers.

Pg 3: some studies reanalyzing the 2017 M87 image (e.g. fig 4 of https://arxiv.org/pdf/2511.17706) have noted support for a low-brightness component to the SE of the ring structure. It would be useful if this paper commented on whether or not that structure is seen in the GenDIRECT reconstructions with a larger field of view.

Pg 3: “these results suggest that the sensitivity of image morphology to the assumed field-of-view reported in independent re-analyses is likely an artifact of insufficient aperture coverage or low quality data.” → I am confused by this sentence – I would have thought all imaging re-analyses of the 2017 EHT data were working from the same public release, so the data quality and coverage should be identical. If the authors have reason to believe that the data release being used in their work differs from that used in the cited Miyoshi+ 2022, it would be very useful to elaborate more on that point.
Pg 4: I understand that the authors have focused on reproducing the 2017 and 2018 images in this paper, but it would be useful to compare these results to the recently released EHT images from the 2021 observations as well (https://arxiv.org/pdf/2509.24593).

Pg 4: Was the xsringgauss modeling done in an existing codebased used by the EHTC (e.g. the eht-imaging modeling.py package) or were these models independently implemented in Dynesty?

Pg 5/Figure 2: Some of the differences in the ring diameter between the new results in this paper and the EHT pipelines are likely due to the correction to the ring diameter introduced by changes in the ring width (wider/more blurred rings will have a smaller diameter for the same dataset). This is discussed in EHTC IV, 2019, and in the recent 2021 results paper the diameter results were corrected for this bias (Eq 23 of https://arxiv.org/pdf/2509.24593). It would be useful to discuss this effect in presenting the results here.

Pg 5: It would be useful to add one or two more sentence of detail in summarizing the GRMHD scoring method adopted here. Were GRMHD images blurred before computing the NXCORR? Is this image library the same as used by the EHTC papers?

Pg 13, datasets: It would be useful to provide links to the exact datasets used in the paper in a footnote.

P 15: In RML imaging with closure amplitudes and phases, it is typically better to use the log of the closure amplitudes than the actual amplitudes, as the noise statistics are better behaved in the log form. Is such a treatment necessary in the closure invariant formalism (i.e. in equation 2)?

P 15: The image domain feature extraction section should cite the relevant section of EHT IV, 2019, as these are the same definitions developed there.

P 18: The section on the GRMHD comparison would benefit from more discussion of the method - in particular, more details should be given on the sentence “We compare all synthetic images to the reconstructed M87 ∗ composites in the image-domain using the normalised cross-correlation metric after correcting for the observer position angle and making small adjustments to scale.” It is not immediately clear to me what these corrections are.

P 18: While I agree that Stokes I MAD images are only moderately sensitive to Rhigh, it would be useful to see a comparable analysis of library images on a second value of Rhigh, if possible.

Extended Fig 2: It would be useful to comment in the text on why the quality of the April 25, 2018 images are significantly worse than the April 21 reconstructions. In Extended Data Figure 1, why does the GenDIReCT composite image for this day look significantly better/cleaner than those presented from the individual FOV reconstructions?

(Remarks on code availability)
The code is well documented and installs and runs cleanly; I was able to easily reproduce the example reconstruction from synthetic EHT data. It would be useful if the authors provided example scripts and links to the exact public EHT datasets

they used to produce the results on M87 shown in this paper, potentially in a separate repository.

Referee #4

(Remarks to the Author)
Review of: "Independent confirmation of the M87* black hole shadow with a new interferometric observable"

The paper describes how GenDIReCT is used to produce a new EHT M87* image, producing a high-quality result with fewer assumptions on calibration and initial image than before. To achieve this, the authors use closure relations and an ML approach to solve the deconvolution problem. All in all, the results are highly significant and the paper is of high quality.

The novelty of the paper arises mostly from the fact that the technique is now successfully applied to what is probably one of the most significant radio images in history, with new ring fitting parameters as a result. The technique itself has been described and tested before, including on EHT data.

One point leaves me unconvinced by these results, which leads me to a question for the authors. The method employs ML to infer the image. Just like other methods, this interpolates and extrapolates unmeasured samples in the uv-plane: some assumptions always go in to solve this ill-posed problem. However, GenDIReCT produces an image with a resolution that is higher than the instrumental resolution. This implies that the image can be downsampled and match the observed data equally well. With traditional methods, the model is at least convolved with a Gaussian to reduce the resolution of the image, so that over-interpretation of details below the resolution is not possible. Here, that is not done. While it is not uncommon to show super-resolution model images in technical/method papers, using such images directly for astrophysical interpretation is rarely done, if at all.

While the GenDIReCT method has seen considerable testing, and the authors have described the method and its assumptions very carefully in earlier papers, it did not fully convince me that what the method does is not comparable to taking the original EHT image, and asking an AI to upsample the image. How can one tell that, by fitting the parameters to the image that is made from ML interpolated and extrapolated uv samples, the added accuracy and/or information is not purely due to using ML in the process? Multiple runs on different datasets do not sufficiently confirm the result, since an ML will behave similar each time.

I note that even with this question in mind, the fact that the results show the ring morphology, and can do this without calibration or starting from an ideal model, is a major result.

I have a few very small comments, which are about the 'methods' section.

- On page 13, "EHT datasets on M87*", the authors describe rescaling the short baselines. What does this exactly mean? The paper contains a reference to code here, but I did not find the answer there (easily). Given that closure values are used, the rescaling of specific baselines can break the closure relations and cause unforeseen effects. Is it not possible (and better) to flag the baseline?

Directly after, the sentence starting "Closure invariants are, in general, computed...", is a bit confusing. I think what the authors may try to describe here is the ordering, averaging before or after calculating the closure invariants, but this can be stated more clearly.

- Under Eq 1, the authors state they create "N^2 - 3N + 1" real-valued invariants. An interferometer with one ref element left out has M(M-1)/2 elements, with M=N-1, giving "N^2/2 - 3N/2 + 1" complex values, or twice that for real valued: N^2 - 3N + 2. Is this a mistake, and should the 'one' be a 'two'?

(Remarks on code availability)
I have scanned the repository to see if it provides the algorithm and can be used for reproducing the result, and I think it satisfies this.

Version 1:

Reviewer comments:

Referee #1

(Remarks to the Author)
Review coauthored with another reviewer

(Remarks on code availability)

Referee #2

(Remarks to the Author)

I co-reviewed this manuscript with one of the reviewers who provided the listed reports.

(Remarks on code availability)

Referee #3

(Remarks to the Author)
I thank the authors for their careful revisions. The addition of the image from the 2021 data is excellent, and the revisions throughout the main text and methods section have greatly improved the quality of the manuscript. The revisions to the github repository are also a great improvement, particularly in the inclusion of the data used in the paper as well as the clear example scripts for running and training the models.

The authors have addressed all of my scientific concerns with the manuscript. I do have some suggestions that I believe would further improve the presentation of the manuscript, but I leave it up to the authors on whether or not to take them.

—-------------------
Main suggestions
—-------------

I strongly suggest moving the beam-convolved images in extended data figure 2 to the main text as a second row in figure 1. This will provide a useful comparison to readers familiar with traditional VLBI presentation as well as the presentation in the main EHT papers, and it will naturally display the superresolution factor achieved by GenDIReCT. (If you take this suggestion, as a minor point I would denote the nominal beam insets in fig 1 with dashed lines to distinguish them from the beam insets in the current extended figure 2, since they are not used for the actual convolution.)

I also suggest using a brightness temperature colorbar in figure 1. I understand the GenDIReCT reconstructions do not have an absolute brightness scale – I suggest simply normalizing each image to exactly 0.5 Jy.

The readme in the “paper-m87” branch of the repo has linkscript for reproducing the new 2021 image, but the user would have to modify this script themselves to reproduce the other datasets. I think it would be very nice if you could provide one script/notebook in the repo that reproduced all three epochs in the main figure 1. I appreciate the inclusion of a script for training the models as well as just running the pre-trained model used in the paper.

—-------------------
Minor points:
—-------------------

Line 14: “gravitationally lensed emission” → Gravitational & doppler redshift is as/more important in producing the central brightness depression in GRMHD & analytic models of the image; I would modify to “gravitationally lensed and redshifted emission”

Line 28: “on event horizon scale” → “on event horizon scales”

In Line 28 you introduce the shadow scale in units of the gravitational radius, then jump to the angular scale in the next paragraph. To ease this transition (and because you already introduce the distance D in line 25), I would just start with the shadow angular scale in line 28, 3\sqrt{3} GM/c^2 D

Line 45-46: “some independent analyses demonstrating reconstructions without a clear ring-like structure” → In context of the previous lines, this reads as if the cited Miyoshi papers don’t recover a ring due to their data weighting terms in an RML framework. But as the next few lines make clear, these papers use traditional CLEAN/hybrid mapping, and their methodological choices are different than the data and regularizer weighting choices in RML. Since the end of the paragraph starting in line 46 already more accurately conveys this information about the Miyoshi et al procedure and results, I would simply delete the clause “with some independent analyses demonstrating reconstructions without a clear ring-like structure.”

Line 87: For maximum clarity, I might say “reconstructions of epochs with lower quality datasets” instead of “reconstructions of lower quality datasets”

Line 113: “We apply the image-domain feature extraction method utilised by the EHTC to measure ring properties” → to me this reads as if you use the REx module, but the next paragraph makes clear you use an independent implementation of the same equations. You could say here “We use an independent implementation of the image-domain feature extraction method developed by the EHTC which we provide in the BASIS software package…”

Line 121: For completeness and symmetry with the presentation of the 2017 and 2018 EHT results, it would be nice to include a listing of the new methods added in the 2021 collaboration analysis here. Furthermore, for the COMRADE analysis cited here, I would add a citation to Tiede+ 2026, which lays out the full methodology used in the 2018 and 2021 COMRADE results.

For maximum clarity in the Table 1 caption, I would note that these are from the unconvolved reconstructions (this will be

particularly important if you take my strong advice to include the convolved versions in Figure 1)

Line 469: “closure invariants do not require the log-transformation typically used with closure amplitudes to stabilize uncertainty statistics” → I am still bumping up against this point. The equation 1 defn of the advariants includes a visibility measurement + noise in the denominator, so if the SNR is low I would expect dividing by the noise to mess up the statistics in the same way it does for normal closure amplitudes. I suppose this is offset by having two visibilities in the numerator and choosing a high SNR reference station, but it does seem to me in less ideal scenarios the invariant statistics may not be well behaved, though they probably do remain better behaved than naive closure amplitudes.

Line 637: I would note here again that you have independently implemented the xsringauss model described in the EHTC papers and are not using existing software from the EHTC.

(Remarks on code availability)
The linked public code repository is substantially improved from the initial submission, including clearer READMEs, all used data sets, and example scripts. I suggest to the authors in my report that they consider including a script that produces the results from all three epochs in figure 1.

Referee #4

(Remarks to the Author)
The authors have adequately revised the manuscript and addressed the concerns raised in the review. I therefore recommend the article for publication.

I remain somewhat concerned about fitting ring parameters to a super-resolution image, as there are no observational constraints on the highest spatial-frequency modes. However, the authors correctly point out that the Event Horizon Telescope Collaboration has employed a similar approach, and the manuscript now clearly explains what was done.

(Remarks on code availability)

Open Access This Peer Review File is licensed under a Creative Commons Attribution 4.0 International License, which permits use, sharing, adaptation, distribution and reproduction in any medium or format, as long as you give appropriate credit to the original author(s) and the source, provide a link to the Creative Commons license, and indicate if changes were made.
In cases where reviewers are anonymous, credit should be given to 'Anonymous Referee' and the source.
The images or other third party material in this Peer Review File are included in the article’s Creative Commons license, unless indicated otherwise in a credit line to the material. If material is not included in the article’s Creative Commons license and your intended use is not permitted by statutory regulation or exceeds the permitted use, you will need to obtain permission directly from the copyright holder.

To view a copy of this license, visit https://creativecommons.org/licenses/by/4.0/

Response to Referees

Samuel Lai, Nithyanandan Thyagarajan, O. Ivy Wong, and Foivos Diakogiannis

We thank the referees for taking the time to carefully read our manuscript, providing valuable feedback, comments, and suggestions. The response has helped us identify areas of the manuscript where additional context or more detailed explanation was necessary, and we present our response to each of the referee's suggestions below. We have divided the response into sections by referee report. Most of the content described in this response has been added to the manuscript as Supplementary Material or as modifications to the main text, which are all highlighted in red.

We list the most significant changes below for convenience:

- We have applied our analysis to the **2021 observations of M87*** which have now entered the public domain.
- Our **updated composites** are now created from the pixel-wise median consistently with the individual dataset aggregates. Any analysis that is derived from composites (i.e. estimation of inclination from comparison with GRMHD+GRRT synthetic images), or figures containing composites, have been updated.
- We have uploaded our complementary **Bayesian framework to GitHub**, and significantly **improved the public GenDIReCT repository** by updating the README and adding a specific branch/release for reproducibility of the M87* results.

Contents

1. Referee #1
2. Referee #2
3. Referee #3
4. Referee #4

Referee #1 (Remarks to the Author):

I have reviewed this manuscript, which presents an application of the GenDIReCT imaging algorithm to EHT 2017 and 2018 M87* data, using interferometric closure invariants as the sole data term. The work addresses an important problem and the core methodology is sound. I consider the central claim broadly supportable, but several specific issues require clarification or revision before the paper is suitable for publication in Nature. My detailed comments follow.

A. Summary of the Key Results

The authors apply GenDIReCT, a novel imaging algorithm utilising a conditional latent diffusion model and multi-layer convolutional neural network trained on closure invariants and non-astronomical images, to six epochs of EHT observations of M87* (four nights from April 2017 and two nights from April 2018). The method reconstructs images without requiring explicit gain calibration, instead operating directly on interferometric closure invariants — quantities that are algebraically immune to station-based multiplicative gain errors. The reconstructed images consistently recover a ring-like morphology. Averaging ring diameter measurements across all six epochs yields an angular diameter of 42.9 ± 0.4 μas, from which the authors derive a black hole mass consistent with the EHTC's 2019 estimate. Data-fidelity and image-similarity metrics are reported, with the best-performing epoch (April 11, 2017) achieving χ_CI^2= 1.1 and ρ_NX image-domain similarity scores of 0.98, 0.94, and 0.93 at field-of-view scales of 128×128, 225×225, and 450×450 μas respectively, evaluated against the EHTC's fiducial reconstruction.

B. Originality and Significance

The application of a generative, closure-invariant-based imaging approach to real EHT M87* data represents a meaningful contribution to the field of VLBI imaging. Demonstrating that a method operating without any gain calibration can recover morphological features consistent with the EHTC's calibrated reconstructions is a valuable cross-check, and the methodology is timely given ongoing development of future EHT observations. The GenDIReCT method itself was published in Lai et al. (2025, PASA); the novelty of the present manuscript lies in its application to real M87* data and in the independent confirmation it provides.

The reference list includes Jennison (1958) and Twiss et al. (1960) among refs 20–26, which is appropriate. However, these are cited collectively in a single bracket alongside more recent developments without distinguishing their individual historical contributions. Separately, while the authors do not claim novelty for the closure concept itself, the title's phrasing — "a new interferometric observable" — may be read by a general Nature readership as implying that the observable itself is new, rather than its application within a generative imaging framework. A brief clarification in the introduction distinguishing the long history of closure quantities from the specific novelty of this work would be beneficial.

We have modified the text in the fourth paragraph of the main text to draw attention to the historical contributions from Jennison 1958 and Twiss et al. 1960 individually. Furthermore, we have focused our novelty claim (i.e. in the abstract/first paragraph and throughout) on two things, the unified closure invariants formalism and the generative imaging framework. We hope this clarifies that the "novel interferometric

observable” in the title references the unified closure invariants formalism, rather than traditional closure quantities in general. However, this does mark the first time that closure invariants from the new formalism are used to image M87*.

The claim of independent confirmation is well-framed: the authors present their result as independent both from the EHTC’s calibrated analysis and from alternative morphological interpretations of M87* (e.g. Miyoshi et al. 2022, 2024), by virtue of operating on calibration-immune observables without reference to either group’s methodology. This is a meaningful and clearly defined form of independence that the paper defends credibly.

C. Data and Methodology

The core methodology is well-motivated and the data (EHT 2017 and 2018 M87* observations) are appropriate and well-established. I have several specific concerns about the methodological presentation.

Description of the generative model and architecture. The paper would benefit from a more self-contained description of the conditional latent diffusion model architecture and its training procedure. In particular, the rationale for key architectural choices — such as CNN details, number of attention layers, latent space dimensionality — is not provided. Similarly, the paper states that augmented CIFAR-10 images and simple geometric shapes are included in the training data to improve generalisation to non-astrophysical morphologies, but no example images from this augmented dataset are shown. Including representative training samples would strengthen the reader’s understanding of the ring-morphology agnosticism encoded in the model.

Median image construction and uncertainty quantification. Several aspects of the ensemble aggregation procedure require clarification. First, the total number of diffusion samples drawn per reconstruction is not stated; the authors should specify this explicitly. Second, the rationale for adopting the pixelwise median rather than the mean as the summary reconstruction is not discussed. Other diffusion-based imaging methods in the literature use the posterior mean; the authors should justify this choice, particularly since the median and mean coincide only for symmetric unimodal distributions and may diverge under multimodal posteriors. Third, and relatedly, no pixel-level uncertainty map is presented. The pixelwise standard deviation of the ensemble — a natural and commonly used uncertainty estimate for generative ensembles — is absent. The authors should explain why the standard deviation was not used, and either provide it or justify its omission.

D. Appropriate Use of Statistics and Treatment of Uncertainties

χ_{CI}^2 as a fidelity metric. The reduced chi-squared statistic χ_{CI}^2 measures consistency with the observed closure invariants. A value near unity confirms that the reconstruction is self-consistent in the closure domain but does not verify that the recovered morphology is correct in an absolute sense. The authors should be explicit that $\chi_{CI}^2 \approx 1$ is a necessary but not sufficient condition for a faithful reconstruction.

We added text to the "Metrics" section under "Methods" to be explicit regarding the significance of a near-unity reconstruction in the chi-square metric. While working on this referee comment, we noticed an outdated piece of information in this section. In the refereed version, we had indicated a 5% systematic uncertainty added in quadrature with the estimated thermal noise, but even in the refereed version, the reduced chi2s reported in illustrations no longer include this additional noise factor. Thus, we have modified this statement in the revised manuscript.

ρ_{NX} as a similarity metric. The ρ_{NX} scores reported are cross-correlations between GenDIReCT reconstructions and the EHTC's fiducial images. These measure inter-method consistency, not ground-truth fidelity. The authors should state this distinction clearly: a high ρ_{NX} indicates that GenDIReCT recovers similar features to RML methods, which is informative, but it does not constitute independent ground-truth validation.

We now have text in the "Metrics" section under "Methods" to be extra clear regarding the inter-method consistency aspect of the rho_NX comparison, rather than as an independent ground-truth validation metric.

E. Conclusions: Robustness, Validity, Reliability

The paper's central conclusion — that an independent analysis of EHT M87* data confirms a ring-like black hole shadow morphology with an angular diameter of 42.9 ± 0.4 μas — is well-supported by the presented reconstructions and metrics. The consistency of the ring morphology across all six epochs, and the stability of ρ_{NX} scores across field-of-view scales, provides genuine support for the robustness of the morphological recovery. The method's agreement with the EHTC's fiducial reconstruction, achieved without any gain calibration, is a credible and meaningful result.

The derived black hole mass rests on the provisional adoption of $\alpha \sim 11.0$ from the EHTC, which the authors themselves acknowledge has not been independently calibrated for GenDIReCT. The mass measurement should therefore be presented

explicitly as provisional, with the associated systematic uncertainty acknowledged. The morphological confirmation — the primary scientific contribution of this work — stands independently of this limitation and is the stronger of the two claims.

We address this below as part of the itemised list in F. Suggested Improvements.

F. Suggested Improvements

I request the following revisions:

1. Architecture description and training data examples. Please provide a more complete description of the diffusion model architecture, including the rationale for key design choices. Include representative examples from the augmented CIFAR-10 and geometric shape training datasets to illustrate the morphological diversity of the training distribution.

We have added a new paragraph in the "GenDIReCT imaging" section under "Methods" to discuss the diffusion model architecture and training data, providing a more self-contained description of the imaging algorithm. As part of the new paragraph, we touched on key design choices including the number of UNet layers, types and location of attention blocks, CNN layers, and latent dimensionality. A new Extended Data Figure is also added to include representative examples of augmented CIFAR-10 and geometric shapes.

2. Ensemble aggregation: sample count, choice of median, and uncertainty maps. Please state explicitly the total number of diffusion samples drawn per reconstruction. Provide a justification for the use of the pixelwise median rather than the mean, with reference to the properties of the posterior distribution. Please also provide pixelwise standard deviation maps as a natural uncertainty quantification, or justify their omission and describe what uncertainty estimate is being offered in their place.

The sample count for each aggregate ensemble is dictated by running our trained model in inference mode for 96 GPU-hours on a single NVIDIA-H100. This typically results in several hundred (~300) individual reconstructions, but the exact number varies slightly by data volume. This information is already present in the "GenDIReCT imaging" section under "Methods" of the refereed edition.

We have amended the "GenDIReCT imaging" section to also address each of the other points by the referee. Here, we summarise our response. We draw 4096 diffusion samples per reconstruction, though the result is typically not sensitive to the

exact number, as long as it is sufficiently large. The image below illustrates a test on synthetic data by number of samples drawn.

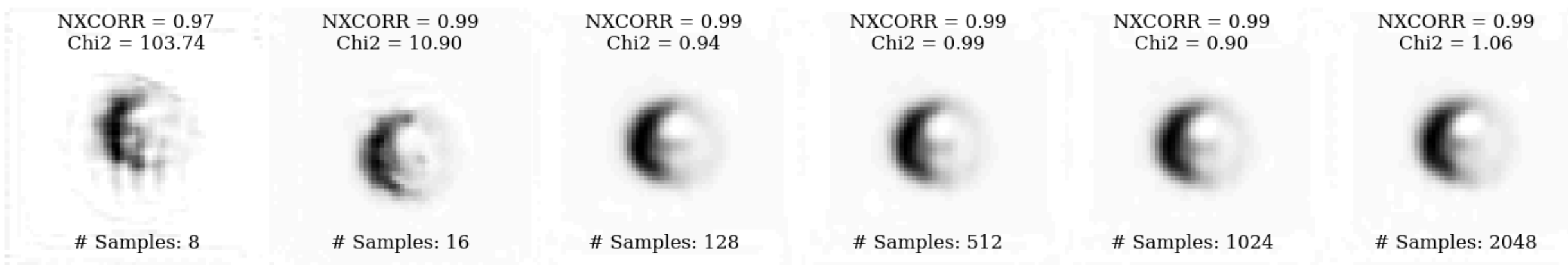

We understand the referee's remarks regarding the posterior mean. In this work, because we use closure-only measurables which do not retain information on absolute position, the alignment of individual reconstruction is a unique problem. We do implement a fairly robust programmatic solution to the alignment issue using the maximum cross-correlation, but some individual outliers have a disproportionate visual impact on the image for the pixel-wise mean. Rather than individually flagging/fixing outliers, we generally find the median to be a sensible aggregate that is more well-behaved, robust to such outliers, and a better representative of the majority of individual reconstructions than the mean. The pixel-wise mean produces a more blended appearance, occasionally with artifacts from alignment outliers, which are not at all representative of individual reconstructions. Note that >50% of reconstructions are usually within the first two most populated and perceptually similar clusters, which helps to justify the pixel-wise median as a statistically sound and accurate aggregate. In the previous (refereed) version, we actually did utilise the pixel-wise mean specifically for constructing composites which used an alternate strategy to bypass the aforementioned problems, but we now adopt the median for the composites as well to remain consistent. Therefore, any figures and downstream analyses that relied on the composites were updated.

We now present our preferred representation of reconstruction confidence in the new updated Extended Data Figure 1. In this and similar works, standard deviation or median absolute deviation (MAD) maps merely outline the ring feature, showing high absolute uncertainty in the ring width, which is expected. We show an example of this in response to another comment below. Instead, we prefer to visualise the inverse relative uncertainty (mean/std or median/MAD), which can be thought of as a signal, or reconstruction confidence map. We have already introduced the use of this map in the context of GenDIReCT in our earlier work (https://ui.adsabs.harvard.edu/abs/2025PASA...42..148L).

3. Provisional mass estimate. The mass estimate should be clearly presented as provisional, with an explicit acknowledgement that the adopted α ~ 11.0 has not been

independently calibrated for GenDIReCT. Where feasible, the authors should propagate the range α = 10.67–12.06 as a systematic uncertainty on the quoted mass.

We have included remarks in the abstract and the main text to further highlight the provisional nature of the mass estimate. In the main text (third paragraph from the end), we have explicitly included the (11.0/alpha) scaling ratio in the black hole mass estimate. Moreover, we have propagated the effect of the full range of alpha calibrations on estimated black hole masses.

Comments on code availability are provided separately in the dedicated code review field.

G. References: Appropriate Credit to Previous Work

The reference list is broadly appropriate. I note two specific concerns. First, refs 20 (Jennison 1958) and 21 (Twiss et al. 1960) are cited collectively within the bracket refs 20–26 without distinguishing their historical roles as the originators of closure phases and closure amplitudes respectively. Given the paper's reference to a "new" observable in the title, explicit historical attribution in the text would strengthen clarity. Second, refs 27 and 28 (Wilkinson et al. 1977; Pearson et al. 1981) are cited to support the claim that closure invariants "underpin high-fidelity morphology reconstructions." These are early VLBI imaging works that do not specifically demonstrate closure-invariant-based reconstruction. More directly relevant citations for this specific claim would be Chael et al. (2018, ref 36) or Thyagarajan et al. (2024, ref 31), which explicitly address imaging from closure quantities.

We have amended the fourth paragraph in the main text to explicitly cite Jennison 1958 and Twiss et al. 1960 separately with reference to their historical roles as originators of closure phases and closure amplitudes respectively. Some of the sentences in these early paragraphs have been rearranged to preserve logical flow to accommodate the change.

For the claim that closure invariants "underpin high-fidelity morphology reconstructions" also in the fourth paragraph of the main text, we have amended the citation list to Chael et al. 2018, Thyagarajan et al. 2024, Lai et al. 2025a, 2025b; the latter three explicitly demonstrate image reconstructions/constraints with closure invariants.

H. Clarity and Context

The abstract and title present the independent confirmation claim clearly, and the paper's overall structure is logical. The precise sense of "independence" — from gain calibration, from the EHTC's methodology, and from alternative morphological interpretations — should be stated explicitly in the abstract and conclusions rather than left implicit.

We have amended the abstract to highlight the calibration independence of our observables and methodological independence.

The paper's description of GenDIReCT as operating "without a prior" warrants revision. The denoising diffusion framework requires sampling from an isotropic Gaussian at the terminal diffusion timestep, which constitutes an explicit prior, and the learned score function encodes an implicit morphological prior from the training distribution. The correct and defensible claim is that GenDIReCT does not require a user-specified, hand-tuned image initialisation at inference time — a genuine and useful property that should be stated accurately.

We identify the conflicting phrase under "Methods" where it was stated that, "our algorithm… does not require an explicit image initialisation or prior". In other parts of the text, we have more carefully and correctly identified the implicit priors from the training distribution and, as the referee pointed out, the initial generation step starts from an isotropic Gaussian noise prior. We decide to remove the "or prior" phrase in the "GenDIReCT imaging" section under "Methods" to focus on the absence of a hand-tuned image initialisation during inference as one of the advantages of the system.

I am willing to reconsider this manuscript following revisions addressing the concerns detailed above.

Referee #1 (Remarks on code availability):

The repository (https://github.com/samlaihei/GenDIReCT) currently presents several barriers to reproduction:
(i) model weights are tracked via Git LFS but this requirement is not documented in the README, causing silent pointer-file checkout and a cryptic UnpicklingError at runtime;
(ii) the ClosureInvariants library installation is not described;
(iii) the image-domain feature extraction code referenced in the paper does not appear to be present in the repository;
(iv) the python scripts are generally not well documented; and
(v) no instructions are provided for reproducing the paper's primary results.

The README should be revised to address the first three points, and the missing code should be included or its absence clearly explained. The python scripts should be documented more comprehensively, and an instruction in README or a Jupyter notebook tutorial should be provided for reproducing the paper's primary results.

Ref. 1, 2, & 3 have remarked that example reproduction scripts and the exact public EHT datasets used would be useful in the public repository. Moreover, Ref. 1 & 2 have made remarks on missing material in the installation instructions. We have improved the installation instructions, added public EHT datasets, included trained model weights of one of the models used in the paper with Git LFS, and added simple example scripts for generating some of the images. The image-domain feature extraction code is packaged with the geometric modelling code, (https://github.com/samlaihei/BASIS), which is also now referenced in the Code Availability section. We copy over this script and demonstrate example usage in the GenDIReCT repository as well.

General improvements to the repository are added to the main branch, but more specific data, models, training script, and basic result analysis code relevant to this paper have been added in a separate branch and release, called "paper-m87". We provide one trained model relevant to the paper and give basic instructions for how one could train a GenDIReCT model, given the provided .uvfits files. At the proofs stage, we will create a DOI for this paper-specific release and replace reference 68, which is currently a generic GenDIReCT v1.0.0 release, without the M87* specific data/models.

Referee #2 (Remarks to the Author):

I co-reviewed this manuscript with one of the reviewers who provided the listed reports.

Referee #3 (Remarks to the Author):

The paper "Independent confirmation of the M87* black hole shadow with a new interferometric observable" presents a new, independent reconstruction of EHT 2017 and 2018 observations of M87* with a novel analysis algorithm GeNDIRECT, which has already been validated and applied to real data in several other papers. In reproducing these important images with a completely independent analysis method, this paper is an important addition to the literature. The approach is well-documented and sound; the underlying method having been validated in several previous papers; the software and underlying data are publicly available. The paper explores uncertainties in the image

reconstructions well, particularly in the Methods section. The paper is overall clear and well presented. However, I have several comments on the presentation and claims throughout that I would like the authors to address.

Pg 1: In paragraph 1, it would help to introduce M87*'s mass and distance in order to quantify the size estimate for the BH shadow image and justify the need to go to mm wavelength VLBI.

We have now introduced M87*'s mass and distance in the first sentence of the first paragraph, which should help put the resolution argument into context.

Pg 1: Since the paper rightly notes that instrument calibration (phase and amplitude) is as significant as sparse u,v coverage in setting the difficulty of the M87* imaging problem, it would be useful to describe these challenges for mm VLBI in one additional sentence in paragraph 2.

We have modified the last sentence of the second paragraph to describe calibration challenges as well. We also modify the surrounding transitional text to accommodate the changes.

Pg 1: In describing the "divergent representations of M87*'s fine-scale features," it would be useful to have a little bit more discussion on what differences between representations are acceptable consequences of solving the ill-posed imaging problem and what are concerning.

We have added a couple of sentences to the third paragraph of the "GenDIReCT imaging" section under "Methods" regarding general expected and unacceptable consequences of the ill-posed imaging problem in the context of diversity in image reconstructions. We figure that this detail is better suited for the "Methods" section than the main text. However, in the third paragraph of the main text, which is the section referenced by the referee, we have added a phrase on "methodological and hyperparameter" sensitivity, which more explicitly points out one of the main sources of the "divergent representations" referenced earlier.

Pg 1: "Iconic ring structure" → I would choose a different adjective here

We have amended the text in the third paragraph. In context, it reads, "with some independent analyses demonstrating reconstructions without a clear ring-like structure". A similar phrase in the abstract was also amended.

Pg 2: I think the paper would benefit from revisiting the summary of closure invariants presented here. The paper says that closure invariants unify the traditional closure phases and amplitudes, but it is not clear from the summary if they carry more information than these data products, which were used in the first EHT papers.

We do specify in the "Methods" section on this point, but we have clarified now in the fourth paragraph of the main text that the closure invariants formalism carries equivalent information to the full set of traditional closure amplitudes plus closure phases with a different form and with a unified treatment. However, the information is packaged in a novel generalised formalism, and this is the first time closure invariants are used to image M87*.

Pg 2: "Unlike other machine-learning based radio interferometric imaging algorithms…." → is the key differences in these other algorithms cited that they use different data products (visibilities or traditional closure quantities) rather than the paper's closure invariants? Or is it the type of prior?

It's true that our approach is the only one to use closure invariants, and it's also true that it's the only one to use conditional latent diffusion, but there are many differences in design philosophy between our approach and other successful approaches that also utilise diffusion models. In order to keep the main text succinct, we have clarified the fifth paragraph of the main text to focus our uniqueness on the closure invariants component. We do not wish to discuss the similarities and differences with other approaches in existing literature in too much detail in the main text.

Pg 2: "we aggregate a large population of reconstructions" → It would be useful to indicate in the main text what parameters in the algorithm determine these different reconstructions.

In the paragraph prior to this sentence (i.e. fifth paragraph of the main text), we introduced GenDIReCT as a trained generative algorithm and highlighted its capabilities of recovering diverse morphological structures without manual intervention, prescriptive priors, or parameter tuning, with more information available in the "Methods" section. When we describe "a large population of reconstructions" here, the reconstructions are generatively produced through the algorithm without tuning any parameters. We make small modifications to the text in both the fifth and sixth paragraphs to emphasise this point.

Fig 1: the main lobe of the PSF/beam is indicated in these panels, but the image is clearly not convolved with it. That is justifiable , but it would be useful to explain that

choice. For comparison to the main EHT result images, it would be useful to also show a version convolved with the same fiducial beam as in those papers.

We now provide an explanation in the "GenDIReCT imaging" section under "Methods". The summary is that like RML and Bayesian methods, the output of GenDIReCT fits the data, while a beam-convolved version would not. GenDIReCT is trained on unconvolved image models and its objective in inference time is to generatively produce image reconstructions consistent with the measurement data. This gives it potential for super-resolution, but structural variations that are nevertheless consistent with the same data would also be recovered generatively. The composites, therefore, are aggregates of all generated reconstructions, which illustrate the most robust and consistently reconstructed features.

However, as suggested by the referee, we now also present 20 uas circular beam-convolved versions in the Extended Data Figures for ease of comparison with the EHT papers. We apply the 20 uas circular beam as it is based on the fiducial DIFMAP resolution. EHT2019 IV shows fiducial reconstructions for each date of observation convolved with this beam in Figure 15.

Pg 3: some studies reanalyzing the 2017 M87 image (e.g. fig 4 of https://arxiv.org/pdf/2511.17706) have noted support for a low-brightness component to the SE of the ring structure. It would be useful if this paper commented on whether or not that structure is seen in the GenDIRECT reconstructions with a larger field of view.

According to the referee's source, the feature is most visibly distinct apart from the main ring in the reconstructions from April 5, 6, 10 or as an extension towards the SE direction on April 11. Based on our aggregate reconstructions and reconstruction confidence maps, as shown in the revised Extended Data Figure 1, there is some evidence for this feature, but the statistical significance is weak. Because our detection is tentative at best, we decide not to comment on this specific feature in this manuscript. However, the inclusion of the reconstruction confidence map enables readers to form their own judgement.

Pg 3: "these results suggest that the sensitivity of image morphology to the assumed field-of-view reported in independent re-analyses is likely an artifact of insufficient aperture coverage or low quality data." → I am confused by this sentence – I would have thought all imaging re-analyses of the 2017 EHT data were working from the same public release, so the data quality and coverage should be identical. If the authors have reason to believe that the data release being used in their work differs from that used in the cited Miyoshi+ 2022, it would be very useful to elaborate more on that point.

The discussed independent re-analyses do work off of the same public datasets. However, not all epochs are of comparable data quality. In this section (i.e. ninth paragraph of the main text), we were following up on an earlier point, which stated that we see some sensitivity to FOV in low-SNR and low uv-coverage datasets (i.e. April 5, 2017), which we do not see in high-quality datasets (i.e. April 11, 2017). In Miyoshi et al. 2022, the comparably lower quality dataset of April 5, 2017 was used to construct the argument in their Sections 5.5 & 5.7 (see also their Figures 24) that, "the EHTC ring appears only when a very narrow BOX is applied." A similar analysis on a higher-quality dataset was not shown. Therefore, we were making a specific point that sensitivity to FOV may arise on datasets of insufficient coverage or quality, but we do not observe the same effect on higher-quality datasets in our methodology. We have modified the text in the ninth paragraph of the main text to enhance clarity.

Pg 4: Was the xsringgauss modeling done in an existing codebased used by the EHTC (e.g. the eht-imaging modeling.py package) or were these models independently implemented in Dynesty?

We have independently implemented the xsringgauss model in a separate framework. To improve the reproducibility of our results, we have made this framework public and provided a reference to it in the Code Availability section (https://github.com/samlaihei/BASIS). Generally, the analysis performed by this framework is not particularly novel as it is functionally identical to the description to EHTC papers; the most novel component is the use of closure invariants alone in the likelihood, which in our tests is preferable over the traditional (log) closure amplitudes and closure phases.

Pg 4: I understand that the authors have focused on reproducing the 2017 and 2018 images in this paper, but it would be useful to compare these results to the recently released EHT images from the 2021 observations as well (https://arxiv.org/pdf/2509.24593).

Now that the 2021 observation datasets have now entered the public domain (https://datacommons.cyverse.org/browse/iplant/home/ckc/EHTC_M87-2018+2021_Jul2026), we have included the same analysis applied to the new epoch. All tables, figures, and relevant text have been updated with the new results.

Pg 5/Figure 2: Some of the differences in the ring diameter between the new results in this paper and the EHT pipelines are likely due to the correction to the ring diameter introduced by changes in the ring width (wider/more blurred rings will have a smaller diameter for the same dataset). This is discussed in EHTC IV, 2019, and in the recent 2021 results paper the diameter results were corrected for this bias (Eq 23 of

https://arxiv.org/pdf/2509.24593). It would be useful to discuss this effect in presenting the results here.

We agree with the referee that this is an important point that warrants careful discussion in the main text, particularly as we have now added the 2021 dataset to our analysis.

As explained by EHT IV 2019, measured diameters from non-CLEAN methods vary smoothly with similar uncertainties, up to about 10%, from no convolution to convolution with a 20 uas beam. Measured diameters from CLEAN-based methods, on the other hand, are heavily influenced by the final kernel convolution procedure, typically applied to blur the point source sky model, with deviations of 20-30%. In order to correct for this effect in general, it's necessary to adopt a forward-modelling procedure to infer the underlying ring diameter, ellipticity, and width simultaneously (i.e. https://arxiv.org/pdf/2509.24593 used a template matching procedure). Though similar in philosophy, this is a fundamentally different procedure in practice compared to the image-domain feature extraction algorithm used in our manuscript and past EHT results. By applying the publicly available EHT CosineRing forward-modelling code (called VIDA) to our composites, we find a ring diameter correction of ~5% at most.

Note that our Bayesian framework is also a forward-modelling approach, albeit with a different geometric template and we forward model closure invariants rather than the image. We recover a consistent mean ring diameter measurement from our Bayesian analysis as the image-domain feature analysis on our non-parametric image reconstructions.

Our preference in this work is to compare the results from all epochs on the same foundation, either all with the correction or without. Unfortunately, uncorrected parameters are not provided for the 2021 epoch and corrected parameters are only presented in https://arxiv.org/pdf/2509.24593 for measurements of each year, rather than each observing day. We also consider that exchanging the more straightforward image-domain feature extraction method with a template-sensitive forward-modelling approach may not necessarily improve interpretability. Hence, across all epochs, we choose to derive diameters directly from the image-domain without further correction/adjustment, consistent with what the EHTC did before the recent publication of the 2021 results. For the 2021 epochs, we recreate and perform image-domain feature extraction on the eht-imaging reconstruction as our sole point of EHTC uncorrected parameter comparison. In the revised main text (seventh paragraph from the end), we now discuss the finite resolution effect. We primarily focus the discussion on the finite resolution effect for the measured ring diameter, as the effect on the

measured ring orientation is minimal and we do not attempt to interpret other measured parameters physically. Accordingly, we have modified our text to present this discussion.

Pg 5: It would be useful to add one or two more sentence of detail in summarizing the GRMHD scoring method adopted here. Were GRMHD images blurred before computing the NXCORR? Is this image library the same as used by the EHTC papers?

We agree with the referee and have added a sentence to explicitly state that the GRMHD images are blurred prior to computing the NXCORR in the final paragraph of the main text. Previously, we blurred by a smaller circular beam, but we have now modified the analysis to blur by the full fitted clean beam, as stated in the text. Generally, the measured NXCORR is higher across the board, and inclinations of max NXCORR have also generally increased, but the observed trends remain consistent.

We do identify our GRMHD simulation library in the “Synthetic M87* images” under “Methods”. It is not exactly the same as the ones used in the EHTC papers, although there is significant overlap in the explored parameter space. We think that this detail is better suited for the “Methods” section, so we have not added text on the image or GRMHD library in the main text.

Pg 13, datasets: It would be useful to provide links to the exact datasets used in the paper in a footnote.

Links to the science-ready observational data underlying this work are provided in the “Data availability” section. We wish to avoid duplicating that footnote, so we have provided the footnote here where indicated by the referee and provided a different, more general link to the EHT Collaboration website in the “Data availability” section. Moreover, we now supply .uvfits files in the code repository for reproducibility.

P 15: In RML imaging with closure amplitudes and phases, it is typically better to use the log of the closure amplitudes than the actual amplitudes, as the noise statistics are better behaved in the log form. Is such a treatment necessary in the closure invariant formalism (i.e. in equation 2)?

By utilizing real and imaginary components in the gauge-theoretic closure invariants formalism, we eliminate the need for differential weighting schemes, and hence an additional hyperparameter, between closure amplitudes and phases, ensuring a consistent treatment of uncertainties. Therefore, there is no natural analogue to the 'log-closure amplitude' transformation within the closure invariant framework. We consider this aspect as an advantage of the closure invariants formalism. In internal

validation tests with *dynesty*, closure invariants as the sole likelihood term was found to be preferable over the independently-weighted log-closure amplitudes and closure phases in terms of convergence and data efficiency. We have added a sentence to the "Closure invariants" section under "Methods" to make this point explicitly.

P 15: The image domain feature extraction section should cite the relevant section of EHT IV, 2019, as these are the same definitions developed there.

We have added one more sentence at the beginning of the "Image-domain feature extraction" section under "Methods" to cite EHT IV, 2019 prior to the discussion of the image-domain feature extraction method.

P 18: The section on the GRMHD comparison would benefit from more discussion of the method - in particular, more details should be given on the sentence "We compare all synthetic images to the reconstructed M87∗ composites in the image-domain using the normalised cross-correlation metric after correcting for the observer position angle and making small adjustments to scale." It is not immediately clear to me what these corrections are.

We have included text in the "Synthetic M87* images" section under "Methods" to clarify further how we have made the comparison between our reconstructions with GRMHD+GRRT synthetic images. In this case, the small adjustments to scale refer to spatial scale. Because ideal GRMHD is scale-agnostic, we chose to rescale our synthetic image library to maximise NXCORR for each parameter combination of spin, inclination, and timestep. Likewise, all synthetic images are fitted with image-domain ring feature extraction to identify the position angle in order to match it with the reconstruction. After adjusting for spatial scale and position angle, they are compared to the composite reconstructions using the NXCORR metric.

P 18: While I agree that Stokes I MAD images are only moderately sensitive to Rhigh, it would be useful to see a comparable analysis of library images on a second value of Rhigh, if possible.

We performed an alternative analysis with Rhigh = 80 and present the result as an Extended Data Figure. Although the inclination of maximum NXCORR is mildly adjusted, the overall spin and inclination trends are near-identical.

Extended Fig 2: It would be useful to comment in the text on why the quality of the April 25, 2018 images are significantly worse than the April 21 reconstructions. In Extended

Data Figure 1, why does the GenDIReCT composite image for this day look significantly better/cleaner than those presented from the individual FOV reconstructions?

The main reasons are the lower data quality and that the LMT station did not participate in observations on April 25, 2018, which introduced a hole in the uv-coverage between 1-2 Glambda in total uv-distance. We note also that the collaboration's Bayesian methods also struggled to convincingly reconstruct a ring on this dataset. We now have some text in the "GenDIReCT imaging" section under "Methods" to discuss this.

The composites are created programmatically for all datasets. In Extended Data Figure 1, the fiducial image shown is the pixelwise median for each individual dataset + FOV. The composite construction does several things differently. First, it considers all individual reconstructions from all FOVs, anchors them to the single best reconstruction (out of all FOVs) with the best data fidelity score, aligns all images to the selected anchor, then takes the median. The difference in appearance is due to the best data fidelity anchor which is in the smallest FOV for April 25, 2018. We added one more phrase to the sixth paragraph in the main text to help clarify this.

Referee #3 (Remarks on code availability):

The code is well documented and installs and runs cleanly; I was able to easily reproduce the example reconstruction from synthetic EHT data. It would be useful if the authors provided example scripts and links to the exact public EHT datasets they used to produce the results on M87 shown in this paper, potentially in a separate repository.

We have created a separate branch titled "paper-m87" which is accompanied by a release. There, we have included data, models, an example training script, and basic result analysis code relevant to this paper. The exact public EHT datasets for the best quality days are present in this branch/release. We provide one trained model relevant to the paper and give basic instructions for how one could train a GenDIReCT model, given the provided .uvfits files. At the proofs stage, we will create a DOI for this paper-specific release and replace reference 68, which is currently pointing to a generic GenDIReCT v1.0.0 release, without the M87* specific data/models.

Referee #4 (Remarks to the Author):

Review of: "Independent confirmation of the M87* black hole shadow with a new interferometric observable"

The paper describes how GenDIReCT is used to produce a new EHT M87* image, producing a high-quality result with fewer assumptions on calibration and initial image than before. To achieve this, the authors use closure relations and an ML approach to solve the deconvolution problem. All in all, the results are highly significant and the paper is of high quality.

The novelty of the paper arises mostly from the fact that the technique is now successfully applied to what is probably one of the most significant radio images in history, with new ring fitting parameters as a result. The technique itself has been described and tested before, including on EHT data.

One point leaves me unconvinced by these results, which leads me to a question for the authors. The method employs ML to infer the image. Just like other methods, this interpolates and extrapolates unmeasured samples in the uv-plane: some assumptions always go in to solve this ill-posed problem. However, GenDIReCT produces an image with a resolution that is higher than the instrumental resolution. This implies that the image can be downsampled and match the observed data equally well. With traditional methods, the model is at least convolved with a Gaussian to reduce the resolution of the image, so that over-interpretation of details below the resolution is not possible. Here, that is not done. While it is not uncommon to show super-resolution model images in technical/method papers, using such images directly for astrophysical interpretation is rarely done, if at all.

We agree with the referee that downsampled versions can match the observed data equally well, implying also that there are many ways for the reconstruction to be "upsampled" by the imaging algorithm. This is one of the benefits of a generative imaging algorithm capable of, in principle, supplying many such solutions, which once aggregated, provides a clearer picture of the most robust reconstructed morphological features. Though we show un-convolved reconstructions, we do, in fact, use beam-convolved versions of the reconstructions when evaluating image-domain similarity as we clarified under the "Metrics" section of "Methods", which is later used for astrophysical interpretation in the downstream inclination analysis. We now show our reconstructions convolved with a 20 uas circular beam for comparison with EHTC fiducial reconstructions. As for image-domain feature extraction, we measure ring characteristics from the direct output of the imaging algorithm without further beam-convolution as the EHTC did with non-CLEAN imaging methods.

While the GenDIReCT method has seen considerable testing, and the authors have described the method and its assumptions very carefully in earlier papers, it did not fully convince me that what the method does is not comparable to taking the original EHT image, and asking an AI to upsample the image. How can one tell that, by fitting the parameters to the image that is made from ML interpolated and extrapolated uv samples, the added accuracy and/or information is not purely due to using ML in the process? Multiple runs on different datasets do not sufficiently confirm the result, since an ML will behave similar each time.

We appreciate the referee's insightful concern. It is important to clarify that GenDIReCT does not utilize image-space priors. Unlike an AI image upscaler which requires an input image to function, our algorithm's output is constrained solely by the provided measurement dataset, or closure invariants in our case. This is a core component of our argument for methodological independence. We also reiterate that we use beam-convolved reconstructions for the downstream inclination analysis, so there should be minimal concern for added accuracy from image interpolation.

Our approach shares the fundamental objective of RML and Bayesian frameworks, that is recovering images consistent with the observational data. These methods are also capable of super-resolution. While RML methods employ explicit regularizers and Bayesian approaches utilize analytical priors, GenDIReCT achieves this with learned weights. We contend that, given that the output from all of these methods fit the data well, the variance in features across these methodologies is scientifically valuable, as it reveals the sensitivity of reconstructed structure to algorithmic assumptions. Ultimately, concordance between GenDIReCT and established techniques serves to identify the most robust physical properties of the source. Simultaneously, we demonstrate a novel, reliable, and complementary solution to recover source morphology from interferometry datasets in the data-sparse and challenging VLBI regime.

I note that even with this question in mind, the fact that the results show the ring morphology, and can do this without calibration or starting from an ideal model, is a major result.

I have a few very small comments, which are about the 'methods' section.

- On page 13, "EHT datasets on M87*", the authors describe rescaling the short baselines. What does this exactly mean? The paper contains a reference to code here, but I did not find the answer there (easily). Given that closure values are used, the rescaling of specific baselines can break the closure relations and cause unforeseen effects. Is it not possible (and better) to flag the baseline?

In this context, the visibility and corresponding uncertainty of very short (effectively zero) baselines are rescaled to a fiducial total flux of 0.6 Jy. The relevant Python code reference is reproduced below.

```
def rescale_zerobaseline(obs, totflux, orig_totflux, uv_max):
    multiplier = totflux / orig_totflux
    for j in range(len(obs.data)):
        if (obs.data['u'][j]**2 + obs.data['v'][j]**2)**0.5 >= uv_max: continue
        for field in ['vis','qvis','uvis','vvis','sigma','qsigma','usigma','vsigma']:
            obs.data[field][j] *= multiplier
```

This rescaling is intentionally a baseline-dependent change as described in EHTC procedures, which influences closure invariants and breaks closure invariance relations for triads that contain the rescaled baseline. However, the impact that it does have on the resulting product (such as excising of some extended flux contribution) is intended by this change. This is part of the imaging pre-processing procedure in some EHT imaging pipelines we have referenced and we intentionally operate under those same conditions.

However, we do test our result on the April 18, 2021 dataset without rescaling and show our result below for the referee’s interest. As shown here, the overall reconstruction morphology is not sensitive to the rescaling procedure, though the minimum chi2 is mildly enhanced. In the below image, the uncertainty map represents the median absolute deviation map and the confidence panel represents the ratio of the median reconstruction over the median absolute deviation.

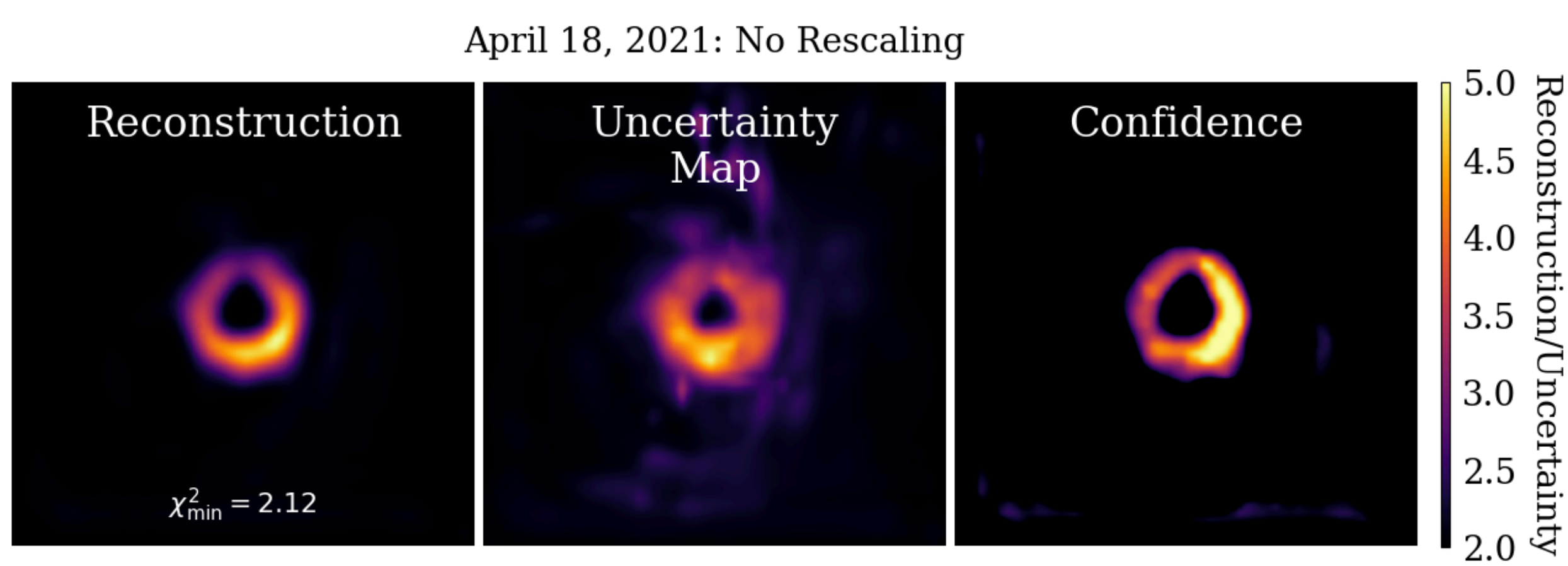

Directly after, the sentence starting "Closure invariants are, in general, computed...", is a bit confusing. I think what the authors may try to describe here is the ordering, averaging before or after calculating the closure invariants, but this can be stated more clearly.

The referee has identified what we intended to convey in this sentence. To enhance intelligibility on first reading, we have rewritten this statement in “EHT datasets on M87*” section under “Methods” with additional clarity. We have also moved this sentence earlier to follow after the sentence on scan-averaging for improved logical flow when reading.

- Under Eq 1, the authors state they create "N^2 - 3N + 1" real-valued invariants. An interferometer with one ref element left out has M(M-1)/2 elements, with M=N-1, giving "N^2/2 - 3N/2 + 1" complex values, or twice that for real valued: N^2 - 3N + 2. Is this a mistake, and should the 'one' be a 'two'?

This is not a typo, but the discrepancy seen here is related to the number of independent degrees of freedom captured by closure quantities. Generally, there are N(N-1) real values from measurements from cross-correlations, with 2N real-valued gains and 1 global phase factor. Removing those degrees of freedom, we have N(N-1) - (2N+1) = N^2 - 3N + 1 maximum degrees of freedom which is exactly the same as the sum of independent closure amplitudes and closure phases.

In the context of our formulation, closure invariants are defined as ratios of advariants formed from triads around a single reference station. The ratio or normalisation component produces the invariant quantities and removes the final degree of freedom in the referee’s combinatorics argument. In other words, there are two real advariants for each of the (N-1)(N-2)/2 independent triangles. By eliminating a common scale factor across all of them, we will get (N-1)(N-2)-1 closure invariants. We refer to Thyagarajan, Nityananda, & Samuel. 2022, “Invariants in copolar interferometry: An Abelian gauge theory” for a more complete explanation of the closure invariants formalism used in this work.

Referee #4 (Remarks on code availability):

I have scanned the repository to see if it provides the algorithm and can be used for reproducing the result, and I think it satisfies this.

Response to Referees

Samuel Lai, Nithyanandan Thyagarajan, O. Ivy Wong, and Foivos Diakogiannis

We thank the referees for taking the time again to carefully read the updated manuscript and recommending the manuscript for acceptance. Based on suggestions from Referee #3, we make a few additional textual and visual modifications to improve the presentation. We further make adjustments to comply with Nature's general guidelines for formatting and file preparation. Below, we copy each of the referee's comments and respond to the suggestions in detail. Additionally, we summarise the main changes to the paper, including modifications made to comply with editorial requirements and formatting guidelines.

In order to keep the number of display items in the Methods section to 10 or below, we have made the following changes:

- **Main text Figure 1 is redesigned** to include the convolved versions, previously in the Methods section (**Extended Data Figure 2 is now removed**).
- **Extended Data Figures 3, 4 and 5 merged into a single grid**. To accomplish this, we kept only the two most populated clusters. The old design is added to the GenDIReCT GitHub repository, which is cited.
- **Extended Data Figure 6 and 7 merged** into one figure.
- **Extended Data Figure 8 and 9 merged** into a single grid.
- **Removal of Extended Data Figure 10**, which demonstrates the image-domain feature extraction technique. This image is similar to plots shown in the EHTC papers which is cited. The refereed version of this plot is instead provided in the GenDIReCT GitHub repository.
- **Posterior covariance plots are merged**, following the same design as Fig. 3 in the main text. Combined plots are Extended Data Figures 12+13, 14+15, and 16 with Fig. 4 of the main text (**Fig. 4 in the main text is therefore removed**).
- **Removal of Extended Data Figure 17,** which showed the image-domain comparative analysis for Rhigh=80, demonstrating insensitivity of the inclination results on the coupled electron temperature model. This was added to demonstrate the robustness of our results to the referee, but given the space constraints of the Extended Data Figures, we decided to leave it out and comment on the experiment in the text instead. The removed figure is added to the GenDIReCT GitHub repository, which is cited.

To aid conciseness in the central narrative of the main text, we have moved several paragraphs containing technical detail to the Methods section. Additionally, we also moved some of the references to the Methods section in order to limit the number of references in the main text to 50. There is some new text in place of the removed paragraphs to summarise the relocated content.

Referee #1, #2 (Remarks to the Author):

I have reviewed the revised manuscript. The authors have responded thoroughly and in good faith to all concerns raised in my previous report. I am satisfied that all issues have been addressed and recommend the manuscript for acceptance.

Referee #1, #2 (Remarks on code availability):

The authors have added all instructions needed to install the required libraries and run the provided code. The requested data, trained model, and training scripts specific to this paper are now provided in a separate branch, whose DOI will replace the one currently referenced.

Referee #3 (Remarks to the Author):

I thank the authors for their careful revisions. The addition of the image from the 2021 data is excellent, and the revisions throughout the main text and methods section have greatly improved the quality of the manuscript. The revisions to the github repository are also a great improvement, particularly in the inclusion of the data used in the paper as well as the clear example scripts for running and training the models.

The authors have addressed all of my scientific concerns with the manuscript. I do have some suggestions that I believe would further improve the presentation of the manuscript, but I leave it up to the authors on whether or not to take them.

—------------------
Main suggestions
—------------

I strongly suggest moving the beam-convolved images in extended data figure 2 to the main text as a second row in figure 1. This will provide a useful comparison to readers familiar with traditional VLBI presentation as well as the presentation in the main EHT papers, and it will naturally display the superresolution factor achieved by GenDIReCT. (If you take this suggestion, as a minor point I would denote the nominal beam insets in fig 1 with dashed lines to distinguish them from the beam insets in the current extended figure 2, since they are not used for the actual convolution.)

I also suggest using a brightness temperature colorbar in figure 1. I understand the GenDIReCT reconstructions do not have an absolute brightness scale – I suggest simply normalizing each image to exactly 0.5 Jy.

We appreciate the suggestion to move the beam-convolved images to Figure 1 as a secondary row and to change the colorbar into a brightness temperature scale. We now show the unconvolved images and the same images convolved with a 20 uas circular beam in the second row. As suggested, we display the beam in dashed lines in the top row.

However, we do not introduce an absolute intensity colorbar, because the values would be imposed, rather than directly measured. For the average reader, we wish to highlight the independent, robust recovery of M87*'s morphology rather than precise measurements of the brightness temperature, which should come from other methods. In the caption, we now include a reference to compact flux constraints measured through other means.

The readme in the "paper-m87" branch of the repo has linkscript for reproducing the new 2021 image, but the user would have to modify this script themselves to reproduce the other datasets. I think it would be very nice if you could provide one script/notebook in the repo that reproduced all three epochs in the main figure 1. I appreciate the inclusion of a script for training the models as well as just running the pre-trained model used in the paper.

We have now included example aligned reconstructions from all three epochs, which when stacked, effectively reproduces the result in Figure 1, albeit with a smaller fraction of the data. A release has been created for the newest version and a DOI for the release is cited in the manuscript.

—------------------
Minor points:
—------------------

Line 14: "gravitationally lensed emission" → Gravitational & doppler redshift is as/more important in producing the central brightness depression in GRMHD & analytic models of the image; I would modify to "gravitationally lensed and redshifted emission"

The qualifier "and redshifted" has been added.

Line 28: "on event horizon scale" → "on event horizon scales"

Corrected in the updated manuscript.

In Line 28 you introduce the shadow scale in units of the gravitational radius, then jump to the angular scale in the next paragraph. To ease this transition (and because you already introduce the distance D in line 25), I would just start with the shadow angular scale in line 28, 3\sqrt{3} GM/c^2 D

Modified the equation to represent an angular scale as suggested.

Line 45-46: “some independent analyses demonstrating reconstructions without a clear ring-like structure” → In context of the previous lines, this reads as if the cited Miyoshi papers don’t recover a ring due to their data weighting terms in an RML framework. But as the next few lines make clear, these papers use traditional CLEAN/hybrid mapping, and their methodological choices are different than the data and regularizer weighting choices in RML. Since the end of the paragraph starting in line 46 already more accurately conveys this information about the Miyoshi et al procedure and results, I would simply delete the clause “with some independent analyses demonstrating reconstructions without a clear ring-like structure.”

We agree that the clause can be misleading given the prior context. Therefore, we rearrange the sentence structure to mitigate these issues.

Line 87: For maximum clarity, I might say “reconstructions of epochs with lower quality datasets” instead of “reconstructions of lower quality datasets”

Added the new phrase for clarity.

Line 113: “We apply the image-domain feature extraction method utilised by the EHTC to measure ring properties” → to me this reads as if you use the REx module, but the next paragraph makes clear you use an independent implementation of the same equations. You could say here “We use an independent implementation of the image-domain feature extraction method developed by the EHTC which we provide in the BASIS software package…”

We have now clarified that our implementation is independent, but we prefer not to mention BASIS by name here. We favour providing its name and DOI in the Methods and Code Availability sections.

Line 121: For completeness and symmetry with the presentation of the 2017 and 2018 EHT results, it would be nice to include a listing of the new methods added in the 2021 collaboration analysis here. Furthermore, for the COMRADE analysis cited here, I would add a citation to Tiede+ 2026, which lays out the full methodology used in the 2018 and 2021 COMRADE results.

We have addressed the new methods in the 2021 collaboration analysis and added the new COMRADE reference. We have also moved this paragraph to the Methods section, as it contains technical detail which is not strictly necessary for the paper's central narrative.

For maximum clarity in the Table 1 caption, I would note that these are from the unconvolved reconstructions (this will be particularly important if you take my strong advice to include the convolved versions in Figure 1)

Agreed and included the unconvolved description.

Line 469: "closure invariants do not require the log-transformation typically used with closure amplitudes to stabilize uncertainty statistics" → I am still bumping up against this point. The equation 1 defn of the advariants includes a visibility measurement + noise in the denominator, so if the SNR is low I would expect dividing by the noise to mess up the statistics in the same way it does for normal closure amplitudes. I suppose this is offset by having two visibilities in the numerator and choosing a high SNR reference station, but it does seem to me in less ideal scenarios the invariant statistics may not be well behaved, though they probably do remain better behaved than naive closure amplitudes.

We agree with the referee's astute point that there will be non-Gaussian noise characteristics in advariants, particularly in low SNR cases. There is a strong argument to more deeply investigate the noise characteristics of these generalised closure invariants as defined. We decided to remove this statement, although it is still true as described in the following sentence that we have found closure invariants without log transformation to be an empirically superior data term to the combination of independently weighted (log-)closure amplitudes and phases.

Line 637: I would note here again that you have independently implemented the xsringauss model described in the EHTC papers and are not using existing software from the EHTC.

We have now added a sentence to note again that the implementation is independent.

Referee #3 (Remarks on code availability):

The linked public code repository is substantially improved from the initial submission, including clearer READMEs, all used data sets, and example scripts. I suggest to the

authors in my report that they consider including a script that produces the results from all three epochs in figure 1.

As above, “we have now included example aligned reconstructions from all three epochs, which when stacked, effectively reproduces the result in Figure 1, albeit with a smaller fraction of the data.”

Referee #4 (Remarks to the Author):

The authors have adequately revised the manuscript and addressed the concerns raised in the review. I therefore recommend the article for publication.

I remain somewhat concerned about fitting ring parameters to a super-resolution image, as there are no observational constraints on the highest spatial-frequency modes. However, the authors correctly point out that the Event Horizon Telescope Collaboration has employed a similar approach, and the manuscript now clearly explains what was done.
